# Supplementary material for: Risk factors for SARS-CoV-2 related mortality and hospitalization before vaccination: A meta-analysis
Source: PLOS Glob Public Health. 2022 Nov 2;2(11):e0001187. doi: 10.1371/journal.pgph.0001187 (PMC10021978; doi:10.1371/journal.pgph.0001187)
Supplement: S1 Text — (DOCX) [file pgph.0001187.s001.docx]

**S1 Text. Predetermined search algorithm**

("COVID-19"[Mesh] OR "SARS-CoV-2"[Mesh] OR COVID-19[Title/Abstract] OR COVID 19[Title/Abstract] OR Novel coronavirus[Title/Abstract] OR SARS cov-2[Title/Abstract] OR SARS cov 2[Title/Abstract]) AND ("Risk Factors"[Mesh] OR risk factor[tiab] OR Risk factors[Title/Abstract]) AND ("Death"[Mesh] OR death[Title/Abstract] OR "Mortality"[Mesh] OR mortality[tiab] OR "Hospitalization"[Mesh] OR hospitalization[tiab] OR hospitalizations[tiab]).
