# Supplementary material for: Risk factors for SARS-CoV-2 related mortality and hospitalization before vaccination: A meta-analysis
Source: PLOS Glob Public Health. 2022 Nov 2;2(11):e0001187. doi: 10.1371/journal.pgph.0001187 (PMC10021978; doi:10.1371/journal.pgph.0001187)
Supplement: S4 Fig — (DOCX) [file pgph.0001187.s005.docx]

**S4 Fig. Funnel plot for coronary artery disease (logistic regression) and mortality**
