# Supplementary material for: Risk factors for SARS-CoV-2 related mortality and hospitalization before vaccination: A meta-analysis
Source: PLOS Glob Public Health. 2022 Nov 2;2(11):e0001187. doi: 10.1371/journal.pgph.0001187 (PMC10021978; doi:10.1371/journal.pgph.0001187)

**S6 Fig. Pooled estimates and 95% confidence intervals for risk factors associated with COVID-19 related mortality in studies using logistic regression (red indicates significance)**


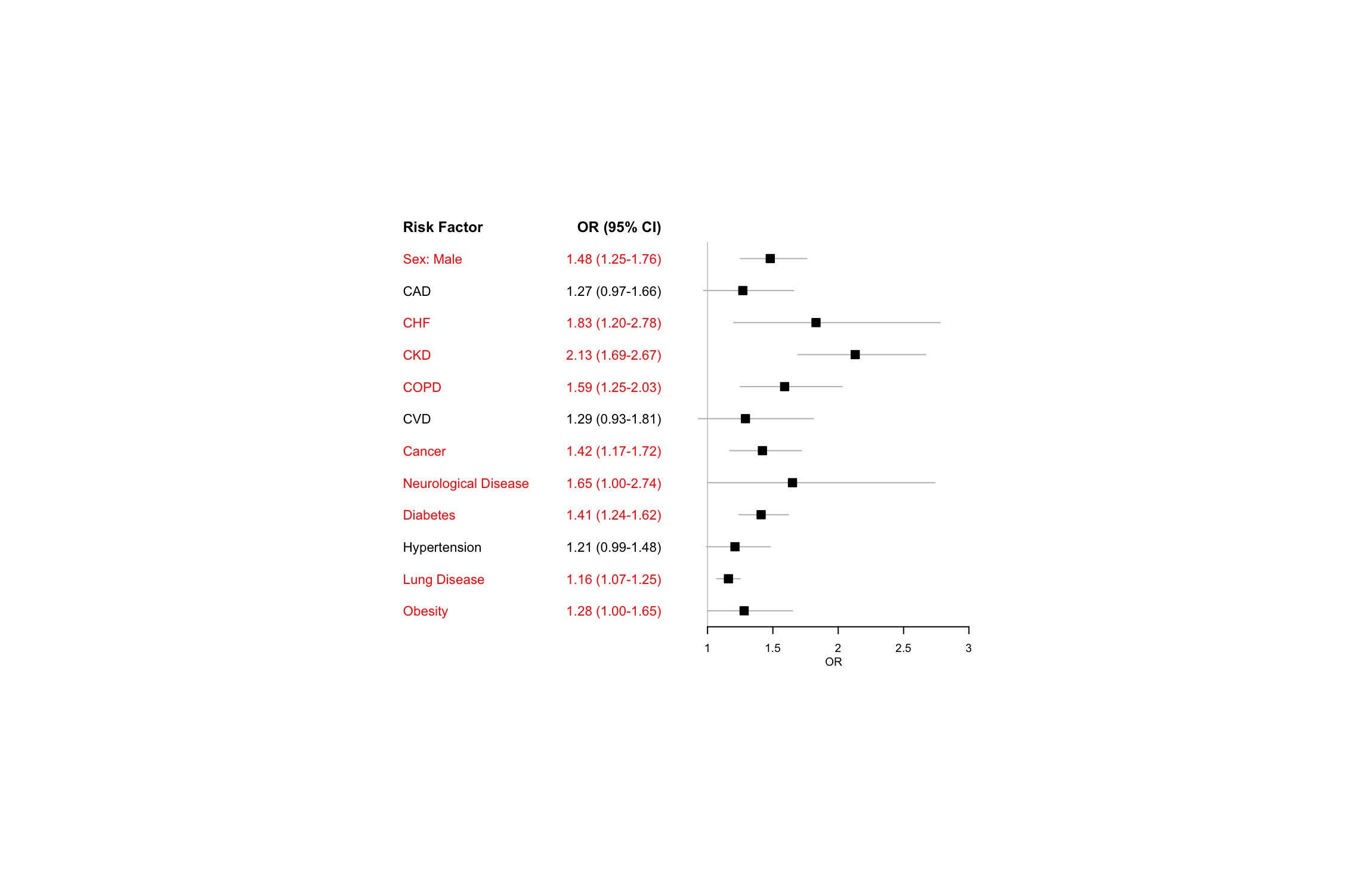

Supplement: S6 Fig — (DOCX) [file pgph.0001187.s007.docx]
