# Supplementary material for: Risk factors for SARS-CoV-2 related mortality and hospitalization before vaccination: A meta-analysis
Source: PLOS Glob Public Health. 2022 Nov 2;2(11):e0001187. doi: 10.1371/journal.pgph.0001187 (PMC10021978; doi:10.1371/journal.pgph.0001187)
Supplement: S1 Table — (DOCX) [file pgph.0001187.s010.docx]

| **S1 Table. Countries included in systematic review** | | |
| --- | --- | --- |
| **Country** | **Mortality**  **(n=71)** | **Hospitalization**  **(n=22)** |
| United States | 26 | 10 |
| Africa | 1 | 0 |
| Bangladesh | 1 | 0 |
| Belgium | 2 | 0 |
| Bolivia | 1 | 0 |
| Brazil | 1 | 1 |
| China | 1 | 0 |
| Croatia | 1 | 0 |
| Democratic Republic of Congo | 1 | 0 |
| Denmark | 1 | 1 |
| Eastern Sudan | 1 | 0 |
| Ecuador | 1 | 0 |
| England | 1 | 2 |
| France | 2 | 0 |
| Germany | 1 | 0 |
| Honduras | 0 | 1 |
| India | 3 | 0 |
| Indonesia | 1 | 0 |
| Iran | 5 | 0 |
| Ireland | 1 | 0 |
| Israel | 0 | 1 |
| Italy | 4 | 1 |
| Korea | 1 | 0 |
| Kuwait | 1 | 0 |
| Mexico | 1 | 2 |
| Nigeria | 1 | 0 |
| Norway | 1 | 1 |
| Pakistan | 1 | 0 |
| Peru | 1 | 0 |
| Portugal | 1 | 1 |
| Russia | 1 | 0 |
| Saudi Arabia | 1 | 0 |
| South Africa | 1 | 0 |
| Spain | 1 | 1 |
| Sweden | 1 | 0 |
| Turkey | 2 | 0 |
