# Supplementary material for: Risk factors for SARS-CoV-2 related mortality and hospitalization before vaccination: A meta-analysis
Source: PLOS Glob Public Health. 2022 Nov 2;2(11):e0001187. doi: 10.1371/journal.pgph.0001187 (PMC10021978; doi:10.1371/journal.pgph.0001187)
Supplement: S2 Table — (DOCX) [file pgph.0001187.s011.docx]

| **S2 Table. Study locations within the United States** | | |
| --- | --- | --- |
| **Location** | **Mortality** | **Hospitalization** |
| California | 1 | 1 |
| Illinois | 2 | 1 |
| Louisiana | 1 | 1 |
| Michigan | 1 | 1 |
| Ohio | 1 | 1 |
| Pennsylvania | 1 | 1 |
| Georgia | 2 | 1 |
| Alabama | 1 | 0 |
| New York | 9 | 1 |
| New Jersey | 2 | 0 |
| Connecticut | 2 | 1 |
| Massachusetts | 2 | 0 |
| Maryland | 1 | 0 |
| Wisconsin | 1 | 1 |
| Washington, D.C. | 1 | 0 |
