# Supplementary material for: Risk factors for SARS-CoV-2 related mortality and hospitalization before vaccination: A meta-analysis
Source: PLOS Glob Public Health. 2022 Nov 2;2(11):e0001187. doi: 10.1371/journal.pgph.0001187 (PMC10021978; doi:10.1371/journal.pgph.0001187)
Supplement: S3 Table — (DOCX) [file pgph.0001187.s012.docx]

**S3 Table. Raw data extracted from individual mortality studies**

| **pmid** | **study** | **adj** | **estimate** | **lb** | **ub** | **measure** | **factor** | **category** | **outcome** | **est meth** | **cov num** |
| --- | --- | --- | --- | --- | --- | --- | --- | --- | --- | --- | --- |
| 32444366 | Petrilli, et al. | 0 | 1.23 | 0.99 | 1.50 | OR | Diabetes |  | mortality | Logistic | 0 |
| 32444366 | Petrilli, et al. | 0 | 1.11 | 0.85 | 1.50 | OR | BMI | 30-39.9 | mortality | Logistic | 0 |
| 32444366 | Petrilli, et al. | 0 | 1.32 | 0.93 | 1.90 | OR | Age | 55-64 | mortality | Logistic | 0 |
| 32444366 | Petrilli, et al. | 0 | 1.06 | 0.85 | 1.30 | OR | Sex | Male | mortality | Logistic | 0 |
| 32444366 | Petrilli, et al. | 0 | 1.42 | 1.08 | 1.90 | OR | Smoking | Unknown | mortality | Logistic | 0 |
| 32444366 | Petrilli, et al. | 0 | 1.05 | 0.54 | 2.00 | OR | BMI | Unknown | mortality | Logistic | 0 |
| 32444366 | Petrilli, et al. | 0 | 0.78 | 0.54 | 1.10 | OR | Age | 45-54 | mortality | Logistic | 0 |
| 32444366 | Petrilli, et al. | 0 | 1.73 | 1.19 | 2.50 | OR | Age | 65-74 | mortality | Logistic | 0 |
| 32444366 | Petrilli, et al. | 0 | 0.94 | 0.73 | 1.20 | OR | BMI | 25-29.9 | mortality | Logistic | 0 |
| 32444366 | Petrilli, et al. | 0 | 1.71 | 1.10 | 2.70 | OR | BMI | >40 | mortality | Logistic | 0 |
| 32444366 | Petrilli, et al. | 0 | 1.93 | 1.40 | 2.60 | OR | CHF |  | mortality | Logistic | 0 |
| 32444366 | Petrilli, et al. | 0 | 0.82 | 0.53 | 1.30 | OR | Smoking | Current | mortality | Logistic | 0 |
| 32444366 | Petrilli, et al. | 0 | 0.92 | 0.71 | 1.20 | OR | CAD |  | mortality | Logistic | 0 |
| 32444366 | Petrilli, et al. | 0 | 1.30 | 0.95 | 1.80 | OR | Cancer |  | mortality | Logistic | 0 |
| 32444366 | Petrilli, et al. | 0 | 2.32 | 1.57 | 3.40 | OR | Age | >75 | mortality | Logistic | 0 |
| 32444366 | Petrilli, et al. | 0 | 0.96 | 0.75 | 1.20 | OR | HTN |  | mortality | Logistic | 0 |
| 32444366 | Petrilli, et al. | 0 | 1.05 | 0.82 | 1.30 | OR | Smoking | Former | mortality | Logistic | 0 |
| 32444366 | Petrilli, et al. | 0 | 0.93 | 0.75 | 1.20 | OR | Pulmonary comorbidity |  | mortality | Logistic | 0 |
| 32444366 | Petrilli, et al. | 0 | 0.73 | 0.55 | 1.00 | OR | CKD |  | mortality | Logistic | 0 |
| 32444366 | Petrilli, et al. | 0 | 0.99 | 0.76 | 1.30 | OR | Asthma |  | mortality | Logistic | 0 |
| 32470210 | Hajifathalian K, et al. | 0 | 1.15 | 0.62 | 2.14 | RR | Obesity |  | mortality | Linear | 0 |
| 32470210 | Hajifathalian K, et al. | 0 | 1.06 | 1.04 | 1.08 | RR | Age |  | mortality | Linear | 0 |
| 32474598 | Bello-Chavolla, et al. | 0 | 1.27 | 1.07 | 1.51 | HR | Immunosuppressed |  | mortality | Cox | 0 |
| 32474598 | Bello-Chavolla, et al. | 0 | 1.40 | 1.23 | 1.59 | HR | COPD |  | mortality | Cox | 0 |
| 32474598 | Bello-Chavolla, et al. | 0 | 1.34 | 1.26 | 1.43 | HR | Diabetes |  | mortality | Cox | 0 |
| 32474598 | Bello-Chavolla, et al. | 0 | 1.25 | 1.17 | 1.34 | HR | Obesity |  | mortality | Cox | 0 |
| 32474598 | Bello-Chavolla, et al. | 0 | 0.26 | 0.23 | 0.29 | HR | Age | <40 | mortality | Cox | 0 |
| 32474598 | Bello-Chavolla, et al. | 0 | 2.02 | 1.89 | 2.16 | HR | Age | >65 | mortality | Cox | 0 |
| 32474598 | Bello-Chavolla, et al. | 0 | 1.99 | 1.77 | 2.23 | HR | CKD |  | mortality | Cox | 0 |
| 32487789 | Escalera-Antezana, et al. | 0 | 9.40 | 1.80 | 104.10 | OR | Age | >60 | mortality | Logistic | 0 |
| 32487789 | Escalera-Antezana, et al. | 0 | 3.30 | 1.30 | 6.30 | OR | HTN |  | mortality | Logistic | 0 |
| 32496252 | Tambe, et al. | 0 | 16.80 | 7.00 | 40.10 | OR | Comorbidity | 1 | mortality | Logistic | 0 |
| 32496259 | Mishra, et al. | 1 | 0.80 | 0.30 | 1.90 | HR | Sex | female | mortality | Cox | 2 |
| 32496259 | Mishra, et al. | 1 | 11.90 | 3.50 | 40.60 | HR | Age | >=60 | mortality | Cox | 2 |
| 32537662 | Rath, et al. | 1 | 3.65 | 1.06 | 12.63 | HR | Diabetes |  | mortality | Cox | 2 |
| 32546725 | Chen, et al. | 0 | 2.59 | 1.58 | 4.26 | HR | Cancer |  | mortality | Cox | 0 |
| 32546725 | Chen, et al. | 0 | 0.35 | 0.26 | 0.48 | HR | Sex | Female | mortality | Cox | 0 |
| 32546725 | Chen, et al. | 0 | 2.21 | 1.68 | 2.90 | HR | HTN |  | mortality | Cox | 0 |
| 32546725 | Chen, et al. | 0 | 1.04 | 1.03 | 1.06 | HR | Age |  | mortality | Cox | 0 |
| 32546725 | Chen, et al. | 0 | 2.56 | 1.90 | 3.45 | HR | CVD |  | mortality | Cox | 0 |
| 32546725 | Chen, et al. | 0 | 2.47 | 1.82 | 3.34 | HR | Diabetes |  | mortality | Cox | 0 |
| 32546725 | Chen, et al. | 0 | 1.84 | 1.17 | 2.92 | HR | Smoking | Current/Former | mortality | Cox | 0 |
| 32589784 | Pettit, et al. | 1 | 5.20 | 1.60 | 16.50 | OR | Sex | male | mortality | Logistic | 11 |
| 32589784 | Pettit, et al. | 1 | 0.30 | 0.03 | 2.90 | OR | CKD |  | mortality | Logistic | 11 |
| 32589784 | Pettit, et al. | 1 | 1.70 | 1.10 | 2.80 | OR | BMI | >=30 | mortality | Logistic | 11 |
| 32589784 | Pettit, et al. | 1 | 0.30 | 0.10 | 0.90 | OR | HTN |  | mortality | Logistic | 11 |
| 32589784 | Pettit, et al. | 1 | 0.70 | 0.20 | 2.30 | OR | CVD |  | mortality | Logistic | 11 |
| 32589784 | Pettit, et al. | 1 | 1.40 | 0.40 | 4.60 | OR | Lung Disease |  | mortality | Logistic | 11 |
| 32589784 | Pettit, et al. | 1 | 0.50 | 0.20 | 1.70 | OR | Diabetes |  | mortality | Logistic | 11 |
| 32589784 | Pettit, et al. | 1 | 0.90 | 0.10 | 6.50 | OR | Stroke |  | mortality | Logistic | 11 |
| 32589784 | Pettit, et al. | 1 | 1.70 | 0.40 | 7.00 | OR | HLD |  | mortality | Logistic | 11 |
| 32589784 | Pettit, et al. | 1 | 3.60 | 2.00 | 6.30 | OR | Age |  | mortality | Logistic | 11 |
| 32589784 | Pettit, et al. | 1 | 2.70 | 0.70 | 10.90 | OR | Cancer |  | mortality | Logistic | 11 |
| 32607513 | Mendy, et al. | 1 | 0.82 | 0.33 | 2.01 | OR | Smoking | Ever | mortality | Logistic | 4 |
| 32607513 | Mendy, et al. | 1 | 1.36 | 0.58 | 3.17 | OR | Sex | Male | mortality | Logistic | 4 |
| 32607513 | Mendy, et al. | 1 | 1.09 | 0.36 | 3.33 | OR | Obesity |  | mortality | Logistic | 4 |
| 32607513 | Mendy, et al. | 1 | 1.79 | 0.75 | 4.27 | OR | Diabetes |  | mortality | Logistic | 4 |
| 32607513 | Mendy, et al. | 1 | 4.48 | 1.81 | 11.08 | OR | CKD |  | mortality | Logistic | 4 |
| 32607513 | Mendy, et al. | 1 | 1.29 | 0.40 | 4.13 | OR | COPD |  | mortality | Logistic | 4 |
| 32607513 | Mendy, et al. | 1 | 1.94 | 1.47 | 2.58 | OR | Age | per 10 year increase | mortality | Logistic | 4 |
| 32607513 | Mendy, et al. | 1 | 1.06 | 0.42 | 2.66 | OR | Cancer | Neoplasm | mortality | Logistic | 4 |
| 32620056 | Shah, et al. | 1 | 3.36 | 1.30 | 8.60 | OR | HTN |  | mortality | Logistic | 14 |
| 32620056 | Shah, et al. | 1 | 3.10 | 1.70 | 5.60 | OR | Age | >=65 | mortality | Logistic | 14 |
| 32620056 | Shah, et al. | 1 | 1.51 | 0.90 | 2.55 | OR | Diabetes |  | mortality | Logistic | 14 |
| 32620056 | Shah, et al. | 1 | 1.08 | 0.51 | 2.28 | OR | CKD |  | mortality | Logistic | 14 |
| 32620056 | Shah, et al. | 1 | 1.03 | 0.55 | 1.93 | OR | Smoking |  | mortality | Logistic | 14 |
| 32620056 | Shah, et al. | 1 | 1.48 | 0.65 | 3.34 | OR | COPD |  | mortality | Logistic | 14 |
| 32620056 | Shah, et al. | 1 | 1.49 | 0.79 | 2.77 | OR | BMI | 30-40 | mortality | Logistic | 14 |
| 32620056 | Shah, et al. | 1 | 3.60 | 1.52 | 8.47 | OR | Immunosuppressed |  | mortality | Logistic | 14 |
| 32620056 | Shah, et al. | 1 | 1.58 | 0.81 | 3.10 | OR | HF |  | mortality | Logistic | 14 |
| 32620056 | Shah, et al. | 1 | 0.74 | 0.33 | 1.64 | OR | Asthma |  | mortality | Logistic | 14 |
| 32620056 | Shah, et al. | 1 | 1.17 | 0.54 | 2.50 | OR | CAD |  | mortality | Logistic | 14 |
| 32620056 | Shah, et al. | 1 | 1.89 | 0.23 | 15.44 | OR | CLD |  | mortality | Logistic | 14 |
| 32620056 | Shah, et al. | 1 | 2.29 | 1.11 | 4.69 | OR | BMI | >=40 | mortality | Logistic | 14 |
| 32620056 | Shah, et al. | 1 | 0.41 | 0.24 | 0.70 | OR | Sex | female | mortality | Logistic | 14 |
| 32620056 | Shah, et al. | 1 | 0.48 | 0.20 | 1.10 | OR | Cancer |  | mortality | Logistic | 14 |
| 32640463 | Williamson, et al. | 1 | 1.15 | 1.05 | 1.27 | HR | Cancer |  | mortality | Cox | 19 |
| 32640463 | Williamson, et al. | 1 | 1.92 | 1.72 | 2.13 | HR | BMI | >40 | mortality | Cox | 19 |
| 32640463 | Williamson, et al. | 1 | 0.89 | 0.85 | 0.93 | HR | HTN |  | mortality | Cox | 19 |
| 32640463 | Williamson, et al. | 1 | 6.08 | 5.52 | 6.69 | HR | Age | 70<80 | mortality | Cox | 19 |
| 32640463 | Williamson, et al. | 1 | 2.16 | 2.03 | 2.27 | HR | Stroke |  | mortality | Cox | 19 |
| 32640463 | Williamson, et al. | 1 | 1.13 | 1.01 | 1.26 | HR | Asthma |  | mortality | Cox | 19 |
| 32640463 | Williamson, et al. | 1 | 1.62 | 1.39 | 1.88 | HR | Hematologic |  | mortality | Cox | 19 |
| 32640463 | Williamson, et al. | 1 | 20.61 | 18.72 | 22.70 | HR | Age | 80+ | mortality | Cox | 19 |
| 32640463 | Williamson, et al. | 1 | 1.19 | 1.11 | 1.27 | HR | Immunosuppressed |  | mortality | Cox | 19 |
| 32640463 | Williamson, et al. | 1 | 2.58 | 2.38 | 2.79 | HR | Neurologic disease |  | mortality | Cox | 19 |
| 32640463 | Williamson, et al. | 1 | 1.59 | 1.53 | 1.65 | HR | Sex | Male | mortality | Cox | 19 |
| 32640463 | Williamson, et al. | 1 | 1.31 | 1.24 | 1.37 | HR | Diabetes |  | mortality | Cox | 19 |
| 32640463 | Williamson, et al. | 1 | 0.99 | 0.93 | 1.05 | HR | Asthma |  | mortality | Cox | 19 |
| 32640463 | Williamson, et al. | 1 | 1.90 | 1.72 | 2.09 | HR | Diabetes |  | mortality | Cox | 19 |
| 32640463 | Williamson, et al. | 1 | 0.96 | 0.91 | 1.03 | HR | Cancer |  | mortality | Cox | 19 |
| 32640463 | Williamson, et al. | 1 | 2.47 | 2.06 | 2.96 | HR | Hematologic |  | mortality | Cox | 19 |
| 32640463 | Williamson, et al. | 1 | 2.40 | 2.16 | 2.66 | HR | Age | 60<70 | mortality | Cox | 19 |
| 32640463 | Williamson, et al. | 1 | 1.75 | 1.51 | 2.03 | HR | CLD |  | mortality | Cox | 19 |
| 32640463 | Williamson, et al. | 1 | 0.89 | 0.82 | 0.97 | HR | Smoking | Current | mortality | Cox | 19 |
| 32640463 | Williamson, et al. | 1 | 0.30 | 0.25 | 0.36 | HR | Age | 40<50 | mortality | Cox | 19 |
| 32640463 | Williamson, et al. | 1 | 1.70 | 1.34 | 2.16 | HR | Immunosuppressed |  | mortality | Cox | 19 |
| 32640463 | Williamson, et al. | 1 | 1.95 | 1.83 | 2.07 | HR | Diabetes |  | mortality | Cox | 19 |
| 32640463 | Williamson, et al. | 1 | 2.52 | 2.33 | 2.72 | HR | CKD |  | mortality | Cox | 19 |
| 32640463 | Williamson, et al. | 1 | 2.82 | 2.09 | 3.81 | HR | Hematologic |  | mortality | Cox | 19 |
| 32640463 | Williamson, et al. | 1 | 1.05 | 1.00 | 1.11 | HR | BMI | 30-34.9 | mortality | Cox | 19 |
| 32640463 | Williamson, et al. | 1 | 1.17 | 1.12 | 1.22 | HR | CHD |  | mortality | Cox | 19 |
| 32640463 | Williamson, et al. | 1 | 1.33 | 1.28 | 1.40 | HR | CKD |  | mortality | Cox | 19 |
| 32640463 | Williamson, et al. | 1 | 1.19 | 1.14 | 1.24 | HR | Smoking | Former | mortality | Cox | 19 |
| 32640463 | Williamson, et al. | 1 | 1.72 | 1.50 | 1.97 | HR | Cancer |  | mortality | Cox | 19 |
| 32640463 | Williamson, et al. | 1 | 1.63 | 1.55 | 1.71 | HR | Pulmonary comorbidity |  | mortality | Cox | 19 |
| 32640463 | Williamson, et al. | 1 | 1.40 | 1.30 | 1.52 | HR | BMI | 35-39.9 | mortality | Cox | 19 |
| 32710646 | Harmouch, et al. | 0 | 1.54 | 0.94 | 2.53 | OR | Smoking |  | mortality | Logistic | 15 |
| 32710646 | Harmouch, et al. | 0 | 2.05 | 1.25 | 3.35 | OR | HTN |  | mortality | Logistic | 15 |
| 32710646 | Harmouch, et al. | 0 | 0.69 | 0.28 | 1.66 | OR | Asthma |  | mortality | Logistic | 15 |
| 32710646 | Harmouch, et al. | 0 | 0.98 | 0.95 | 1.01 | OR | BMI |  | mortality | Logistic | 15 |
| 32710646 | Harmouch, et al. | 0 | 3.78 | 2.28 | 6.25 | OR | CKD |  | mortality | Logistic | 15 |
| 32710646 | Harmouch, et al. | 0 | 1.28 | 0.80 | 2.06 | OR | Sex | male | mortality | Logistic | 15 |
| 32710646 | Harmouch, et al. | 0 | 3.89 | 2.09 | 7.22 | OR | CHF |  | mortality | Logistic | 15 |
| 32710646 | Harmouch, et al. | 0 | 3.30 | 1.58 | 6.90 | OR | Vascular disease |  | mortality | Logistic | 15 |
| 32710646 | Harmouch, et al. | 0 | 2.44 | 0.75 | 7.96 | OR | Cancer |  | mortality | Logistic | 15 |
| 32710646 | Harmouch, et al. | 0 | 4.18 | 1.45 | 12.08 | OR | ESRD |  | mortality | Logistic | 15 |
| 32710646 | Harmouch, et al. | 0 | 2.21 | 1.09 | 4.46 | OR | Lung Disease |  | mortality | Logistic | 15 |
| 32710646 | Harmouch, et al. | 0 | 1.04 | 0.59 | 1.83 | OR | Obesity |  | mortality | Logistic | 15 |
| 32710646 | Harmouch, et al. | 0 | 0.90 | 0.55 | 1.48 | OR | Diabetes |  | mortality | Logistic | 15 |
| 32710646 | Harmouch, et al. | 0 | 0.73 | 0.17 | 3.25 | OR | Immunosuppressed |  | mortality | Logistic | 15 |
| 32710646 | Harmouch, et al. | 0 | 1.07 | 1.05 | 1.08 | OR | Age |  | mortality | Logistic | 15 |
| 32712623 | Nakeshbandi, et al. | 1 | 1.10 | 0.92 | 1.30 | RR | Diabetes |  | mortality |  | 5 |
| 32712623 | Nakeshbandi, et al. | 1 | 1.30 | 1.00 | 1.70 | RR | BMI | >=30 | mortality |  | 5 |
| 32712623 | Nakeshbandi, et al. | 1 | 2.70 | 1.00 | 6.90 | RR | Age | 45-64 | mortality |  | 5 |
| 32712623 | Nakeshbandi, et al. | 1 | 1.40 | 1.10 | 1.90 | RR | BMI | 25-29.99 | mortality |  | 5 |
| 32712623 | Nakeshbandi, et al. | 1 | 0.81 | 0.66 | 1.00 | RR | HTN |  | mortality |  | 5 |
| 32712623 | Nakeshbandi, et al. | 1 | 4.50 | 1.80 | 11.00 | RR | Age | >=65 | mortality |  | 5 |
| 32712623 | Nakeshbandi, et al. | 1 | 1.30 | 1.00 | 1.50 | RR | Sex | male | mortality |  | 5 |
| 32722159 | Nogueira, et al. | 1 | 0.75 | 0.55 | 1.01 | OR | Diabetes |  | hospitalization + mortality | Logistic | 12 |
| 32722159 | Nogueira, et al. | 1 | 0.54 | 0.13 | 1.51 | OR | Asthma |  | hospitalization + mortality | Logistic | 12 |
| 32722159 | Nogueira, et al. | 1 | 2.95 | 2.16 | 4.00 | OR | CKD |  | hospitalization + mortality | Logistic | 12 |
| 32722159 | Nogueira, et al. | 1 | 0.88 | 0.35 | 1.94 | OR | CLD |  | hospitalization + mortality | Logistic | 12 |
| 32722159 | Nogueira, et al. | 1 | 1.21 | 0.73 | 1.95 | OR | hematological disorder |  | hospitalization + mortality | Logistic | 12 |
| 32722159 | Nogueira, et al. | 1 | 1.58 | 1.17 | 2.14 | OR | neuromuscular disorder |  | hospitalization + mortality | Logistic | 12 |
| 32722159 | Nogueira, et al. | 1 | 2.86 | 1.51 | 5.32 | OR | Heart disease |  | hospitalization + mortality | Logistic | 12 |
| 32722159 | Nogueira, et al. | 1 | 20.36 | 10.59 | 41.31 | OR | Age | 66-70 | hospitalization + mortality | Logistic | 12 |
| 32722159 | Nogueira, et al. | 1 | 50.91 | 28.46 | 98.50 | OR | Age | 76-80 | hospitalization + mortality | Logistic | 12 |
| 32722159 | Nogueira, et al. | 1 | 6.01 | 2.68 | 13.40 | OR | Age | 56-60 | hospitalization + mortality | Logistic | 12 |
| 32722159 | Nogueira, et al. | 1 | 1.47 | 1.20 | 1.79 | OR | Sex | male | hospitalization + mortality | Logistic | 12 |
| 32722159 | Nogueira, et al. | 1 | 91.83 | 51.10 | 178.31 | OR | Age | 91-95 | hospitalization + mortality | Logistic | 12 |
| 32722159 | Nogueira, et al. | 1 | 0.92 | 0.64 | 1.32 | OR | Cancer |  | hospitalization + mortality | Logistic | 12 |
| 32722159 | Nogueira, et al. | 1 | 1.48 | 0.53 | 3.52 | OR | HIV |  | hospitalization + mortality | Logistic | 12 |
| 32722159 | Nogueira, et al. | 1 | 70.65 | 40.35 | 134.69 | OR | Age | 81-85 | hospitalization + mortality | Logistic | 12 |
| 32722159 | Nogueira, et al. | 1 | 34.01 | 18.64 | 66.69 | OR | Age | 71-75 | hospitalization + mortality | Logistic | 12 |
| 32722159 | Nogueira, et al. | 1 | 10.50 | 5.12 | 22.17 | OR | Age | 61-65 | hospitalization + mortality | Logistic | 12 |
| 32722159 | Nogueira, et al. | 1 | 83.23 | 47.51 | 58.70 | OR | Age | 86-90 | hospitalization + mortality | Logistic | 12 |
| 32722159 | Nogueira, et al. | 1 | 140.17 | 70.69 | 291.53 | OR | Age | 96-104 | hospitalization + mortality | Logistic | 12 |
| 32722159 | Nogueira, et al. | 1 | 1.05 | 0.75 | 1.47 | OR | Lung Disease |  | hospitalization + mortality | Logistic | 12 |
| 32726242 | Smith, et al. | 1 | 1.43 | 1.07 | 1.92 | RR | Diabetes |  | mortality | Linear | 4 |
| 32726242 | Smith, et al. | 1 | 4.70 | 2.40 | 9.12 | RR | Age | >=60 | mortality | Linear | 4 |
| 32726242 | Smith, et al. | 1 | 1.41 | 1.06 | 1.88 | RR | COPD |  | mortality | Linear | 4 |
| 32726242 | Smith, et al. | 1 | 0.75 | 0.57 | 0.98 | RR | HLD |  | mortality | Linear | 4 |
| 32730358 | Zhao, et al. | 0 | 9.23 | 1.90 | 45.01 | OR | COPD |  | mortality | Logistic | 3 |
| 32730358 | Zhao, et al. | 0 | 4.90 | 1.17 | 20.50 | OR | Age | >63 | mortality | Logistic | 3 |
| 32730358 | Zhao, et al. | 0 | 33.48 | 4.99 | 224.45 | OR | HF |  | mortality | Logistic | 3 |
| 32743602 | McPadden J, et al. | 0 | 1.03 | 0.74 | 1.43 | OR | cardiac arrythmias |  | hospitalization + mortality | Logistic | 0 |
| 32743602 | McPadden J, et al. | 0 | 23.34 | 10.06 | 64.09 | OR | Age | 85+ | hospitalization + mortality | Logistic | 0 |
| 32743602 | McPadden J, et al. | 0 | 1.32 | 0.88 | 1.94 | OR | CLD |  | hospitalization + mortality | Logistic | 0 |
| 32743602 | McPadden J, et al. | 0 | 0.94 | 0.67 | 1.33 | OR | PVD |  | hospitalization + mortality | Logistic | 0 |
| 32743602 | McPadden J, et al. | 0 | 1.01 | 0.64 | 1.55 | OR | RA/collagen vascular disease |  | hospitalization + mortality | Logistic | 0 |
| 32743602 | McPadden J, et al. | 0 | 1.01 | 0.74 | 1.39 | OR | Lung Disease |  | hospitalization + mortality | Logistic | 0 |
| 32743602 | McPadden J, et al. | 0 | 1.06 | 0.71 | 1.56 | OR | CHF |  | hospitalization + mortality | Logistic | 0 |
| 32743602 | McPadden J, et al. | 0 | 1.15 | 0.67 | 1.93 | OR | Cancer |  | hospitalization + mortality | Logistic | 0 |
| 32743602 | McPadden J, et al. | 0 | 1.10 | 0.73 | 1.67 | OR | HTN, uncomplicated |  | hospitalization + mortality | Logistic | 0 |
| 32743602 | McPadden J, et al. | 0 | 1.10 | 0.72 | 1.68 | OR | diabetes, complicated |  | hospitalization + mortality | Logistic | 0 |
| 32743602 | McPadden J, et al. | 0 | 0.84 | 0.57 | 1.21 | OR | solid tumor |  | hospitalization + mortality | Logistic | 0 |
| 32743602 | McPadden J, et al. | 0 | 1.76 | 1.33 | 2.35 | OR | Sex | male | hospitalization + mortality | Logistic | 0 |
| 32743602 | McPadden J, et al. | 0 | 0.88 | 0.56 | 1.36 | OR | HTN, complicated |  | hospitalization + mortality | Logistic | 0 |
| 32743602 | McPadden J, et al. | 0 | 1.22 | 0.89 | 1.69 | OR | BMI | "obesity" | hospitalization + mortality | Logistic | 0 |
| 32743602 | McPadden J, et al. | 0 | 0.78 | 0.56 | 1.08 | OR | depression |  | hospitalization + mortality | Logistic | 0 |
| 32743602 | McPadden J, et al. | 0 | 4.20 | 1.83 | 11.38 | OR | Age | 55-64 | hospitalization + mortality | Logistic | 0 |
| 32743602 | McPadden J, et al. | 0 | 15.66 | 6.91 | 42.34 | OR | Age | 75-84 | hospitalization + mortality | Logistic | 0 |
| 32743602 | McPadden J, et al. | 0 | 7.10 | 3.13 | 19.16 | OR | Age | 65-74 | hospitalization + mortality | Logistic | 0 |
| 32743602 | McPadden J, et al. | 0 | 1.31 | 0.89 | 1.93 | OR | CKD |  | hospitalization + mortality | Logistic | 0 |
| 32743602 | McPadden J, et al. | 0 | 1.78 | 0.63 | 5.42 | OR | Age | 45-54 | hospitalization + mortality | Logistic | 0 |
| 32743602 | McPadden J, et al. | 0 | 0.75 | 0.53 | 1.06 | OR | valvular disease |  | hospitalization + mortality | Logistic | 0 |
| 32743602 | McPadden J, et al. | 0 | 1.00 | 0.27 | 2.84 | OR | HIV |  | hospitalization + mortality | Logistic | 0 |
| 32743602 | McPadden J, et al. | 0 | 1.21 | 0.39 | 3.32 | OR | lymphoma |  | hospitalization + mortality | Logistic | 0 |
| 32743602 | McPadden J, et al. | 0 | 1.47 | 1.06 | 2.05 | OR | Neurologic disease |  | hospitalization + mortality | Logistic | 0 |
| 32743602 | McPadden J, et al. | 0 | 0.99 | 0.66 | 1.47 | OR | diabetes, uncomplicated |  | hospitalization + mortality | Logistic | 0 |
| 32743602 | McPadden J, et al. | 0 | 1.12 | 0.81 | 1.54 | OR | hypothyroidism |  | hospitalization + mortality | Logistic | 0 |
| 32744714 | De Vito, et al | 1 | 0.71 | 0.17 | 3.06 | OR | Age | >72 | mortality | Logistic | 3 |
| 32744714 | De Vito, et al | 1 | 0.47 | 0.11 | 2.00 | OR | HTN |  | mortality | Logistic | 3 |
| 32744714 | De Vito, et al | 1 | 0.67 | 0.18 | 2.44 | OR | COPD |  | mortality | Logistic | 3 |
| 32766546 | Almazeedi S, et al. | 1 | 2.09 | 0.27 | 16.08 | OR | CKD |  | mortality | Logistic | 7 |
| 32766546 | Almazeedi S, et al. | 1 | 0.22 | 0.03 | 1.51 | OR | BMI | >=30 | mortality | Logistic | 7 |
| 32766546 | Almazeedi S, et al. | 1 | 10.09 | 1.22 | 83.40 | OR | Smoking |  | mortality | Logistic | 7 |
| 32766546 | Almazeedi S, et al. | 1 | 0.84 | 0.46 | 4.81 | OR | HTN |  | mortality | Logistic | 7 |
| 32766546 | Almazeedi S, et al. | 1 | 3.03 | 0.58 | 15.81 | OR | Age | >50 | mortality | Logistic | 7 |
| 32766546 | Almazeedi S, et al. | 1 | 0.83 | 0.17 | 4.16 | OR | Diabetes |  | mortality | Logistic | 7 |
| 32766546 | Almazeedi S, et al. | 1 | 4.92 | 1.03 | 23.44 | OR | Asthma |  | mortality | Logistic | 7 |
| 32783686 | Tartof, et al. | 1 | 1.38 | 0.93 | 2.06 | RR | HTN |  | death | Poisson | 16 |
| 32783686 | Tartof, et al. | 1 | 1.26 | 0.82 | 1.95 | RR | BMI | 30-34 | death | Poisson | 16 |
| 32783686 | Tartof, et al. | 1 | 1.17 | 0.83 | 1.65 | RR | CKD |  | death | Poisson | 16 |
| 32783686 | Tartof, et al. | 1 | 0.84 | 0.30 | 2.35 | RR | Smoking | current | death | Poisson | 16 |
| 32783686 | Tartof, et al. | 1 | 1.88 | 0.71 | 4.95 | RR | Age | 41-50 | death | Poisson | 16 |
| 32783686 | Tartof, et al. | 1 | 1.31 | 0.89 | 1.91 | RR | PVD |  | death | Poisson | 16 |
| 32783686 | Tartof, et al. | 1 | 43.21 | 17.80 | 104.92 | RR | Age | >=80 | death | Poisson | 16 |
| 32783686 | Tartof, et al. | 1 | 1.16 | 0.63 | 2.17 | RR | BMI | 35-39 | death | Poisson | 16 |
| 32783686 | Tartof, et al. | 1 | 0.91 | 0.62 | 1.35 | RR | BMI | 25-29 | death | Poisson | 16 |
| 32783686 | Tartof, et al. | 1 | 1.66 | 1.04 | 2.64 | RR | MI |  | death | Poisson | 16 |
| 32783686 | Tartof, et al. | 1 | 3.14 | 1.31 | 7.57 | RR | Age | 51-60 | death | Poisson | 16 |
| 32783686 | Tartof, et al. | 1 | 16.08 | 6.72 | 38.52 | RR | Age | 71-80 | death | Poisson | 16 |
| 32783686 | Tartof, et al. | 1 | 1.06 | 0.64 | 1.75 | RR | Cancer |  | death | Poisson | 16 |
| 32783686 | Tartof, et al. | 1 | 1.44 | 0.92 | 2.25 | RR | Immunosuppressed |  | death | Poisson | 16 |
| 32783686 | Tartof, et al. | 1 | 1.37 | 0.93 | 2.03 | RR | cerebrovasc disease |  | death | Poisson | 16 |
| 32783686 | Tartof, et al. | 1 | 2.68 | 1.43 | 5.04 | RR | BMI | 40-44 | death | Poisson | 16 |
| 32783686 | Tartof, et al. | 1 | 0.81 | 0.54 | 1.21 | RR | Asthma |  | death | Poisson | 16 |
| 32783686 | Tartof, et al. | 1 | 0.84 | 0.56 | 1.27 | RR | CHF |  | death | Poisson | 16 |
| 32783686 | Tartof, et al. | 1 | 1.81 | 0.99 | 3.30 | RR | BMI | <18.5 | death | Poisson | 16 |
| 32783686 | Tartof, et al. | 1 | 1.30 | 0.96 | 1.75 | RR | Smoking | former | death | Poisson | 16 |
| 32783686 | Tartof, et al. | 1 | 4.18 | 2.12 | 8.26 | RR | BMI | >=45 | death | Poisson | 16 |
| 32783686 | Tartof, et al. | 1 | 1.47 | 1.02 | 2.11 | RR | HLD |  | death | Poisson | 16 |
| 32783686 | Tartof, et al. | 1 | 7.18 | 3.08 | 16.78 | RR | Age | 61-70 | death | Poisson | 16 |
| 32783686 | Tartof, et al. | 1 | 1.50 | 1.12 | 2.01 | RR | Sex | male | death | Poisson | 16 |
| 32783686 | Tartof, et al. | 1 | 6.54 | 2.66 | 16.12 | RR | organ transplant |  | death | Poisson | 16 |
| 32783686 | Tartof, et al. | 1 | 0.93 | 0.60 | 1.42 | RR | Lung Disease |  | death | Poisson | 16 |
| 32810610 | Vena, et al. | 1 | 1.07 | 1.04 | 1.10 | OR | Age |  | mortality | Logistic | 2 |
| 32810610 | Vena, et al. | 1 | 2.58 | 1.07 | 6.25 | OR | CVD |  | mortality | Logistic | 2 |
| 32815621 | Czernichow, et al. | 1 | 1.85 | 1.27 | 2.79 | OR | Age | 45-64 | mortality | Logistic | 11 |
| 32815621 | Czernichow, et al. | 1 | 0.97 | 0.74 | 1.27 | OR | HLD |  | mortality | Logistic | 11 |
| 32815621 | Czernichow, et al. | 1 | 1.65 | 1.32 | 2.07 | OR | Cancer |  | mortality | Logistic | 11 |
| 32815621 | Czernichow, et al. | 1 | 2.79 | 1.95 | 3.97 | OR | BMI | 35-39.9 | mortality | Logistic | 11 |
| 32815621 | Czernichow, et al. | 1 | 1.01 | 0.82 | 1.24 | OR | HTN |  | mortality | Logistic | 11 |
| 32815621 | Czernichow, et al. | 1 | 1.24 | 0.77 | 1.93 | OR | BMI | <18.5 | mortality | Logistic | 11 |
| 32815621 | Czernichow, et al. | 1 | 1.15 | 0.82 | 1.59 | OR | HF |  | mortality | Logistic | 11 |
| 32815621 | Czernichow, et al. | 1 | 1.41 | 1.12 | 1.77 | OR | BMI | 25-29.9 | mortality | Logistic | 11 |
| 32815621 | Czernichow, et al. | 1 | 1.34 | 1.11 | 1.62 | OR | Diabetes |  | mortality | Logistic | 11 |
| 32815621 | Czernichow, et al. | 1 | 1.04 | 0.74 | 1.45 | OR | OSA |  | mortality | Logistic | 11 |
| 32815621 | Czernichow, et al. | 1 | 1.73 | 1.35 | 2.21 | OR | CKD |  | mortality | Logistic | 11 |
| 32815621 | Czernichow, et al. | 1 | 1.49 | 1.22 | 1.81 | OR | Sex | male | mortality | Logistic | 11 |
| 32815621 | Czernichow, et al. | 1 | 1.89 | 1.45 | 2.47 | OR | BMI | 30-34.9 | mortality | Logistic | 11 |
| 32815621 | Czernichow, et al. | 1 | 5.28 | 3.63 | 7.93 | OR | Age | 65-79 | mortality | Logistic | 11 |
| 32815621 | Czernichow, et al. | 1 | 1.06 | 0.84 | 1.33 | OR | Smoking |  | mortality | Logistic | 11 |
| 32815621 | Czernichow, et al. | 1 | 2.55 | 1.62 | 3.95 | OR | BMI | 40+ | mortality | Logistic | 11 |
| 32853230 | Rossi, et al. | 1 | 1.20 | 0.60 | 2.20 | HR | Vascular disease |  | hospitalization + Mortality | Cox | 14 |
| 32853230 | Rossi, et al. | 1 | 1.50 | 0.90 | 2.60 | HR | CKD |  | hospitalization + Mortality | Cox | 14 |
| 32853230 | Rossi, et al. | 1 | 1.80 | 1.30 | 2.50 | HR | arrythmia |  | hospitalization + Mortality | Cox | 14 |
| 32853230 | Rossi, et al. | 1 | 1.30 | 0.60 | 2.90 | HR | Obesity |  | hospitalization + Mortality | Cox | 14 |
| 32853230 | Rossi, et al. | 0 | 1.60 | 1.20 | 2.10 | HR | Sex | male | hospitalization + Mortality | Cox | 14 |
| 32853230 | Rossi, et al. | 1 | 2.30 | 1.60 | 3.20 | HR | HF |  | hospitalization + Mortality | Cox | 14 |
| 32853230 | Rossi, et al. | 0 | 27.80 | 12.50 | 61.70 | HR | Age | >=81 | hospitalization + Mortality | Cox | 14 |
| 32853230 | Rossi, et al. | 1 | 1.60 | 1.10 | 2.20 | HR | Diabetes |  | hospitalization + Mortality | Cox | 14 |
| 32853230 | Rossi, et al. | 0 | 3.80 | 1.60 | 9.40 | HR | Age | 61-70 | hospitalization + Mortality | Cox | 14 |
| 32853230 | Rossi, et al. | 0 | 1.50 | 0.50 | 4.20 | HR | Age | 51-60 | hospitalization + Mortality | Cox | 14 |
| 32853230 | Rossi, et al. | 1 | 1.70 | 1.20 | 2.50 | HR | CHD |  | hospitalization + Mortality | Cox | 14 |
| 32853230 | Rossi, et al. | 0 | 9.10 | 4.00 | 20.60 | HR | Age | 71-80 | hospitalization + Mortality | Cox | 14 |
| 32853230 | Rossi, et al. | 1 | 1.40 | 0.90 | 2.20 | HR | HLD |  | hospitalization + Mortality | Cox | 14 |
| 32853230 | Rossi, et al. | 1 | 1.10 | 0.70 | 1.70 | HR | COPD |  | hospitalization + Mortality | Cox | 14 |
| 32853230 | Rossi, et al. | 1 | 1.60 | 1.20 | 2.10 | HR | HTN |  | hospitalization + Mortality | Cox | 14 |
| 32853230 | Rossi, et al. | 1 | 1.80 | 1.10 | 2.80 | HR | Dementia |  | hospitalization + Mortality | Cox | 14 |
| 32853230 | Rossi, et al. | 1 | 1.40 | 1.00 | 2.00 | HR | Cancer |  | hospitalization + Mortality | Cox | 14 |
| 32887982 | Reilev, et al. | 1 | 1.90 | 1.40 | 2.60 | OR | CKD |  | mortality | Logistic | 2 |
| 32887982 | Reilev, et al. | 1 | 1.30 | 1.00 | 1.70 | OR | Cancer |  | mortality | Logistic | 2 |
| 32887982 | Reilev, et al. | 1 | 29.90 | 17.20 | 51.90 | OR | Age |  | mortality | Logistic | 3 |
| 32887982 | Reilev, et al. | 1 | 1.40 | 1.10 | 1.80 | OR | Stroke |  | mortality | Logistic | 2 |
| 32887982 | Reilev, et al. | 1 | 2.60 | 1.60 | 4.00 | OR | Comorbidity |  | mortality | Logistic | 2 |
| 32887982 | Reilev, et al. | 1 | 2.00 | 1.50 | 2.60 | OR | Dementia |  | mortality | Logistic | 2 |
| 32887982 | Reilev, et al. | 1 | 2.60 | 1.70 | 4.10 | OR | Comorbidity |  | mortality | Logistic | 2 |
| 32887982 | Reilev, et al. | 1 | 3.50 | 2.20 | 5.40 | OR | Comorbidity |  | mortality | Logistic | 2 |
| 32887982 | Reilev, et al. | 1 | 5.20 | 3.40 | 8.00 | OR | Comorbidity |  | mortality | Logistic | 2 |
| 32887982 | Reilev, et al. | 1 | 4.40 | 2.50 | 7.90 | OR | Age |  | mortality | Logistic | 3 |
| 32887982 | Reilev, et al. | 1 | 1.40 | 1.10 | 1.80 | OR | Lung Disease |  | mortality | Logistic | 2 |
| 32887982 | Reilev, et al. | 1 | 1.50 | 1.10 | 2.00 | OR | Obesity |  | mortality | Logistic | 2 |
| 32887982 | Reilev, et al. | 1 | 15.20 | 8.70 | 26.30 | OR | Age |  | mortality | Logistic | 3 |
| 32887982 | Reilev, et al. | 1 | 90.20 | 50.20 | 162.20 | OR | Age |  | mortality | Logistic | 3 |
| 32887982 | Reilev, et al. | 1 | 2.10 | 1.70 | 2.60 | OR | Sex |  | mortality | Logistic | 3 |
| 32887982 | Reilev, et al. | 1 | 1.80 | 1.30 | 2.40 | OR | HF |  | mortality | Logistic | 2 |
| 32887982 | Reilev, et al. | 1 | 1.30 | 1.10 | 1.60 | OR | HTN |  | mortality | Logistic | 2 |
| 32887982 | Reilev, et al. | 1 | 1.80 | 1.00 | 3.30 | OR | CLD |  | mortality | Logistic | 2 |
| 32887982 | Reilev, et al. | 1 | 1.60 | 1.20 | 2.00 | OR | AF |  | mortality | Logistic | 2 |
| 32887982 | Reilev, et al. | 1 | 1.10 | 0.90 | 1.40 | OR | Heart disease |  | mortality | Logistic | 2 |
| 32887982 | Reilev, et al. | 1 | 1.60 | 1.30 | 2.00 | OR | Diabetes |  | mortality | Logistic | 2 |
| 32892789 | Santos, et al. | 0 | 2.77 | 2.66 | 2.88 | HR | Age |  | mortality | Cox | 0 |
| 32892789 | Santos, et al. | 0 | 1.01 | 0.98 | 1.03 | HR | Sex |  | mortality | Cox | 0 |
| 32892789 | Santos, et al. | 0 | 1.18 | 1.13 | 1.16 | HR | HF |  | mortality | Cox | 0 |
| 32892789 | Santos, et al. | 0 | 0.66 | 0.61 | 0.73 | HR | Asthma |  | mortality | Cox | 0 |
| 32892789 | Santos, et al. | 0 | 1.16 | 0.54 | 2.45 | HR | BMI |  | mortality | Cox | 0 |
| 32892789 | Santos, et al. | 0 | 1.19 | 1.14 | 1.23 | HR | Diabetes |  | mortality | Cox | 0 |
| 32892789 | Santos, et al. | 0 | 1.18 | 0.53 | 2.61 | HR | BMI |  | mortality | Cox | 0 |
| 32892789 | Santos, et al. | 0 | 0.93 | 0.87 | 1.00 | HR | Immunosuppressed |  | mortality | Cox | 0 |
| 32892789 | Santos, et al. | 0 | 1.13 | 1.01 | 1.26 | HR | CLD |  | mortality | Cox | 0 |
| 32892789 | Santos, et al. | 0 | 1.27 | 0.59 | 2.70 | HR | BMI |  | mortality | Cox | 0 |
| 32892789 | Santos, et al. | 0 | 1.74 | 1.67 | 1.82 | HR | Age |  | mortality | Cox | 0 |
| 32892789 | Santos, et al. | 0 | 1.03 | 0.91 | 1.16 | HR | Cancer |  | mortality | Cox | 0 |
| 32892789 | Santos, et al. | 0 | 1.00 | 0.47 | 2.12 | HR | BMI |  | mortality | Cox | 0 |
| 32892789 | Santos, et al. | 0 | 1.31 | 1.24 | 1.39 | HR | Neurologic disease |  | mortality | Cox | 0 |
| 32892789 | Santos, et al. | 0 | 1.25 | 1.19 | 1.32 | HR | CKD |  | mortality | Cox | 0 |
| 32945856 | Miller, et al. | 1 | 1.36 | 1.03 | 1.81 | OR | CHF |  | mortality | Logistic | 17 |
| 32945856 | Miller, et al. | 1 | 1.31 | 1.02 | 1.68 | OR | COPD |  | mortality | Logistic | 17 |
| 32945856 | Miller, et al. | 1 | 0.93 | 0.73 | 1.20 | OR | Obesity |  | mortality | Logistic | 17 |
| 32945856 | Miller, et al. | 1 | 1.28 | 0.90 | 1.82 | OR | Stroke |  | mortality | Logistic | 17 |
| 32945856 | Miller, et al. | 1 | 1.24 | 0.96 | 1.61 | OR | CKD |  | mortality | Logistic | 17 |
| 32945856 | Miller, et al. | 1 | 1.18 | 0.92 | 1.52 | OR | Diabetes |  | mortality | Logistic | 17 |
| 32945856 | Miller, et al. | 1 | 1.06 | 0.76 | 1.49 | OR | Cancer |  | mortality | Logistic | 17 |
| 32945856 | Miller, et al. | 1 | 1.51 | 1.20 | 1.91 | OR | Sex |  | mortality | Logistic | 17 |
| 32945856 | Miller, et al. | 1 | 1.90 | 1.43 | 2.52 | OR | Dementia |  | mortality | Logistic | 17 |
| 32945856 | Miller, et al. | 1 | 0.69 | 0.53 | 0.92 | OR | HTN |  | mortality | Logistic | 17 |
| 32945856 | Miller, et al. | 1 | 1.38 | 0.97 | 1.98 | OR | PVD |  | mortality | Logistic | 17 |
| 32945856 | Miller, et al. | 1 | 2.77 | 1.92 | 4.00 | OR | Age |  | mortality | Logistic | 17 |
| 32945856 | Miller, et al. | 1 | 1.46 | 1.11 | 1.90 | OR | CAD |  | mortality | Logistic | 17 |
| 32960645 | Garibaldi, et al. | 1 | 1.19 | 1.10 | 1.28 | HR | BMI |  | mortality | Cox | 24 |
| 32975574 | Kabarriti, et al. | 1 | 1.21 | 1.05 | 1.40 | HR | Diabetes |  | mortality | Cox | 16 |
| 32975574 | Kabarriti, et al. | 1 | 9.48 | 5.98 | 15.00 | HR | Age | >80 | mortality | Cox | 16 |
| 32975574 | Kabarriti, et al. | 1 | 1.85 | 1.17 | 2.94 | HR | Age | 41-60 | mortality | Cox | 16 |
| 32975574 | Kabarriti, et al. | 1 | 1.54 | 1.32 | 1.80 | HR | CKD |  | mortality | Cox | 16 |
| 32975574 | Kabarriti, et al. | 1 | 1.48 | 1.21 | 1.81 | HR | Dementia |  | mortality | Cox | 16 |
| 32975574 | Kabarriti, et al. | 1 | 1.38 | 1.15 | 1.67 | HR | BMI | >35 | mortality | Cox | 16 |
| 32975574 | Kabarriti, et al. | 1 | 1.02 | 0.88 | 1.19 | HR | HTN |  | mortality | Cox | 16 |
| 32975574 | Kabarriti, et al. | 1 | 5.24 | 3.37 | 8.16 | HR | Age | 61-80 | mortality | Cox | 16 |
| 32975574 | Kabarriti, et al. | 1 | 0.98 | 0.84 | 1.15 | HR | Lung Disease |  | mortality | Cox | 16 |
| 32975574 | Kabarriti, et al. | 1 | 0.72 | 0.62 | 0.83 | HR | Sex | female | mortality | Cox | 16 |
| 32975574 | Kabarriti, et al. | 1 | 1.20 | 1.03 | 1.41 | HR | CVD |  | mortality | Cox | 16 |
| 32975574 | Kabarriti, et al. | 1 | 1.26 | 0.93 | 1.70 | HR | hemi/paraplegia |  | mortality | Cox | 16 |
| 32975574 | Kabarriti, et al. | 1 | 1.23 | 0.99 | 1.52 | HR | CLD |  | mortality | Cox | 16 |
| 32975574 | Kabarriti, et al. | 1 | 0.88 | 0.71 | 1.09 | HR | Cancer |  | mortality | Cox | 16 |
| 32975574 | Kabarriti, et al. | 1 | 0.94 | 0.63 | 1.40 | HR | BMI | <18.5 | mortality | Cox | 16 |
| 32975575 | Munoz-Price, et al. | 1 | 0.05 | 0.01 | 0.43 | OR | Sex | Male | mortality | Logistic | 13 |
| 32975575 | Munoz-Price, et al. | 1 | 1.19 | 1.05 | 1.35 | OR | BMI | unit increase | mortality | Logistic | 13 |
| 32975575 | Munoz-Price, et al. | 1 | 22.79 | 3.38 | 53.81 | OR | Age | >=60 | mortality | Logistic | 13 |
| 32975575 | Munoz-Price, et al. | 1 | 7.15 | 0.55 | 9.22 | OR | Smoking | current or former | mortality | Logistic | 13 |
| 33020114 | Adrish, et al. | 1 | 0.67 | 0.53 | 0.84 | HR | Sex | Female | survival | Cox | 2 |
| 33020114 | Adrish, et al. | 1 | 1.02 | 1.02 | 1.03 | HR | Age |  | survival | Cox | 2 |
| 33020335 | Rozaliyani, et al. | 0 | 4.27 | 3.08 | 5.92 | OR | Diabetes |  | mortality | Logistic | 0 |
| 33020335 | Rozaliyani, et al. | 0 | 1.12 | 0.63 | 1.99 | OR | COPD |  | mortality | Logistic | 0 |
| 33020335 | Rozaliyani, et al. | 0 | 1.91 | 1.52 | 2.39 | OR | Sex | male | mortality | Logistic | 0 |
| 33020335 | Rozaliyani, et al. | 0 | 3.47 | 1.08 | 11.15 | OR | Immunosuppressed |  | mortality | Logistic | 0 |
| 33020335 | Rozaliyani, et al. | 0 | 1.85 | 0.66 | 5.21 | OR | Age | 50-69 | mortality | Logistic | 0 |
| 33020335 | Rozaliyani, et al. | 0 | 0.16 | 0.03 | 0.93 | OR | Age | 10-19 | mortality | Logistic | 0 |
| 33020335 | Rozaliyani, et al. | 0 | 4.63 | 1.61 | 13.33 | OR | Age | >70 | mortality | Logistic | 0 |
| 33020335 | Rozaliyani, et al. | 0 | 5.15 | 3.53 | 7.50 | OR | Heart disease |  | mortality | Logistic | 0 |
| 33020335 | Rozaliyani, et al. | 0 | 2.87 | 0.58 | 14.30 | OR | Cancer |  | mortality | Logistic | 0 |
| 33020335 | Rozaliyani, et al. | 0 | 0.38 | 0.13 | 1.08 | OR | Age | 20-49 | mortality | Logistic | 0 |
| 33020335 | Rozaliyani, et al. | 0 | 5.76 | 0.96 | 34.66 | OR | CLD |  | mortality | Logistic | 0 |
| 33020335 | Rozaliyani, et al. | 0 | 9.77 | 5.05 | 18.91 | OR | CKD |  | mortality | Logistic | 0 |
| 33020335 | Rozaliyani, et al. | 0 | 5.18 | 3.87 | 6.93 | OR | HTN |  | mortality | Logistic | 0 |
| 33035307 | Munblit, et al. | 1 | 2.10 | 1.46 | 2.99 | OR | Diabetes |  | mortality | Logistic | 10 |
| 33035307 | Munblit, et al. | 1 | 1.27 | 0.77 | 2.02 | OR | Lung Disease |  | mortality | Logistic | 10 |
| 33035307 | Munblit, et al. | 1 | 2.73 | 1.34 | 5.47 | OR | Dementia |  | mortality | Logistic | 10 |
| 33035307 | Munblit, et al. | 1 | 1.78 | 1.24 | 2.57 | OR | Heart disease |  | mortality | Logistic | 10 |
| 33035307 | Munblit, et al. | 1 | 1.71 | 1.24 | 2.37 | OR | Sex | male | mortality | Logistic | 10 |
| 33035307 | Munblit, et al. | 1 | 1.09 | 0.63 | 1.81 | OR | Neurologic disease |  | mortality | Logistic | 10 |
| 33035307 | Munblit, et al. | 1 | 1.05 | 1.03 | 1.06 | OR | Age | per 1 yr incr | mortality | Logistic | 10 |
| 33035307 | Munblit, et al. | 1 | 1.70 | 1.14 | 2.59 | OR | HTN |  | mortality | Logistic | 10 |
| 33035307 | Munblit, et al. | 1 | 2.99 | 1.89 | 4.64 | OR | CKD |  | mortality | Logistic | 10 |
| 33035307 | Munblit, et al. | 1 | 1.36 | 0.76 | 2.32 | OR | Cancer |  | mortality | Logistic | 10 |
| 33038592 | Calmes, et al. | 0 | 1.70 | 0.94 | 3.20 | OR | Cancer |  | mortality | Logistic | 0 |
| 33038592 | Calmes, et al. | 0 | 0.59 | 0.20 | 1.80 | OR | Asthma |  | mortality | Logistic | 0 |
| 33038592 | Calmes, et al. | 0 | 1.00 | 0.96 | 1.05 | OR | BMI |  | mortality | Logistic | 0 |
| 33038592 | Calmes, et al. | 0 | 2.50 | 1.20 | 5.30 | OR | CKD |  | mortality | Logistic | 0 |
| 33038592 | Calmes, et al. | 0 | 1.80 | 1.10 | 3.10 | OR | Diabetes |  | mortality | Logistic | 0 |
| 33038592 | Calmes, et al. | 0 | 1.90 | 0.95 | 3.80 | OR | COPD |  | mortality | Logistic | 0 |
| 33038592 | Calmes, et al. | 0 | 2.20 | 1.40 | 3.50 | OR | Sex | male | mortality | Logistic | 0 |
| 33038592 | Calmes, et al. | 0 | 1.30 | 0.68 | 2.30 | OR | emphysema |  | mortality | Logistic | 0 |
| 33038592 | Calmes, et al. | 0 | 1.90 | 1.60 | 2.30 | OR | Age | by ten years | mortality | Logistic | 0 |
| 33038592 | Calmes, et al. | 0 | 1.40 | 0.81 | 2.30 | OR | Smoking |  | mortality | Logistic | 0 |
| 33038592 | Calmes, et al. | 0 | 1.40 | 0.86 | 2.40 | OR | Dyslipidemia |  | mortality | Logistic | 0 |
| 33038592 | Calmes, et al. | 0 | 1.40 | 0.80 | 2.30 | OR | HTN |  | mortality | Logistic | 0 |
| 33038592 | Calmes, et al. | 0 | 3.80 | 1.60 | 8.60 | OR | Immunosuppressed |  | mortality | Logistic | 0 |
| 33038592 | Calmes, et al. | 0 | 0.86 | 0.45 | 1.70 | OR | GERD |  | mortality | Logistic | 0 |
| 33038592 | Calmes, et al. | 0 | 1.80 | 1.10 | 3.20 | OR | Obesity |  | mortality | Logistic | 0 |
| 33038592 | Calmes, et al. | 0 | 1.90 | 1.10 | 3.20 | OR | cardiopathy |  | mortality | Logistic | 0 |
| 33043705 | Ramachandran, et al. | 1 | 1.28 | 0.64 | 2.54 | HR | Smoking |  | mortality | Cox | 4 |
| 33043705 | Ramachandran, et al. | 1 | 2.28 | 1.15 | 4.54 | HR | Cancer |  | mortality | Cox | 4 |
| 33043705 | Ramachandran, et al. | 1 | 1.18 | 0.51 | 2.73 | HR | Age | >60 | mortality | Cox | 4 |
| 33043705 | Ramachandran, et al. | 1 | 0.90 | 0.44 | 1.82 | HR | HTN |  | mortality | Cox | 4 |
| 33051749 | Mohammed, et al. | 0 | 0.99 | 0.58 | 1.67 | OR | Sex | female | mortality | Logistic | 3 |
| 33051749 | Mohammed, et al. | 1 | 9.63 | 3.84 | 24.15 | OR | chronic disease | presence | mortality | Logistic | 3 |
| 33051749 | Mohammed, et al. | 1 | 1.06 | 1.04 | 1.08 | OR | Age |  | mortality | Logistic | 3 |
| 33090436 | Doganci, et al. | 0 | 3.90 | 1.19 | 12.84 | OR | CKD |  | mortality | Logistic | 7 |
| 33090436 | Doganci, et al. | 0 | 1.10 | 1.07 | 1.14 | OR | Age |  | mortality | Logistic | 7 |
| 33090436 | Doganci, et al. | 0 | 8.02 | 3.61 | 17.83 | OR | COPD |  | mortality | Logistic | 7 |
| 33090436 | Doganci, et al. | 0 | 1.92 | 0.93 | 3.95 | OR | HTN |  | mortality | Logistic | 7 |
| 33090436 | Doganci, et al. | 0 | 4.63 | 2.08 | 10.30 | OR | CHD |  | mortality | Logistic | 7 |
| 33090436 | Doganci, et al. | 0 | 1.54 | 0.72 | 3.29 | OR | Diabetes |  | mortality | Logistic | 7 |
| 33090436 | Doganci, et al. | 0 | 22.03 | 6.73 | 72.14 | OR | Cancer |  | mortality | Logistic | 7 |
| 33112411 | Lunski, et al. | 1 | 5.96 | 4.71 | 7.54 | OR | Age | >=65 | mortality | Logistic | 9 |
| 33112411 | Lunski, et al. | 1 | 2.15 | 1.73 | 2.67 | OR | Sex | male | mortality | Logistic | 9 |
| 33112411 | Lunski, et al. | 1 | 1.43 | 0.99 | 2.08 | OR | CAD |  | mortality | Logistic | 9 |
| 33112411 | Lunski, et al. | 1 | 1.30 | 1.03 | 1.63 | OR | BMI | >=30 | mortality | Logistic | 9 |
| 33112411 | Lunski, et al. | 1 | 1.34 | 0.96 | 1.88 | OR | COPD |  | mortality | Logistic | 9 |
| 33112411 | Lunski, et al. | 1 | 0.60 | 0.47 | 0.78 | OR | HTN |  | mortality | Logistic | 9 |
| 33112411 | Lunski, et al. | 1 | 3.84 | 2.98 | 4.93 | OR | CKD |  | mortality | Logistic | 9 |
| 33112411 | Lunski, et al. | 1 | 2.03 | 1.44 | 2.87 | OR | Cancer |  | mortality | Logistic | 9 |
| 33112411 | Lunski, et al. | 1 | 0.92 | 0.70 | 1.21 | OR | Diabetes |  | mortality | Logistic | 9 |
| 33115547 | Islam, et al. | 1 | 7.56 | 3.19 | 17.92 | OR | Age | >=60 | mortality | Logistic + RR | 8 |
| 33115547 | Islam, et al. | 1 | 4.65 |  |  | RR | CHD |  | mortality | Logistic + RR | 8 |
| 33115547 | Islam, et al. | 1 | 2.63 |  |  | RR | Diabetes |  | mortality | Logistic + RR | 8 |
| 33115547 | Islam, et al. | 1 | 9.03 |  |  | RR | CKD |  | mortality | Logistic + RR | 8 |
| 33115547 | Islam, et al. | 1 | 7.30 |  |  | RR | COPD |  | mortality | Logistic + RR | 8 |
| 33115547 | Islam, et al. | 1 | 3.38 |  |  | RR | HTN |  | mortality | Logistic + RR | 8 |
| 33115547 | Islam, et al. | 1 | 10.52 |  |  | RR | CLD |  | mortality | Logistic + RR | 8 |
| 33115547 | Islam, et al. | 1 | 9.73 |  |  | RR | Cancer |  | mortality | Logistic + RR | 8 |
| 33128848 | Kim, et al. | 1 | 0.81 | 0.67 | 0.98 | OR | Asthma |  | mortality | Logistic | 13 |
| 33128848 | Kim, et al. | 1 | 1.20 | 1.08 | 1.32 | OR | Diabetes |  | mortality | Logistic | 13 |
| 33128848 | Kim, et al. | 1 | 2.63 | 2.29 | 3.01 | OR | Age | 60-79 | mortality | Logistic | 13 |
| 33128848 | Kim, et al. | 1 | 0.51 | 0.37 | 0.70 | OR | Age | <40 | mortality | Logistic | 13 |
| 33128848 | Kim, et al. | 1 | 1.15 | 0.97 | 1.35 | OR | Cancer |  | mortality | Logistic | 13 |
| 33128848 | Kim, et al. | 1 | 1.61 | 1.30 | 2.00 | OR | BMI | >=40 | mortality | Logistic | 13 |
| 33128848 | Kim, et al. | 1 | 1.00 | 0.87 | 1.16 | OR | BMI | 30-34.9 | mortality | Logistic | 13 |
| 33128848 | Kim, et al. | 1 | 0.73 | 0.64 | 0.83 | OR | Smoking | active/former | mortality | Logistic | 13 |
| 33128848 | Kim, et al. | 1 | 1.02 | 0.90 | 1.17 | OR | CAD |  | mortality | Logistic | 13 |
| 33128848 | Kim, et al. | 1 | 1.00 |  |  | OR | BMI | 18.5-24.9 | mortality | Logistic | 13 |
| 33128848 | Kim, et al. | 1 | 1.59 | 1.37 | 1.86 | OR | HF |  | mortality | Logistic | 13 |
| 33128848 | Kim, et al. | 1 | 1.44 | 1.08 | 1.92 | OR | BMI | <18.5 | mortality | Logistic | 13 |
| 33128848 | Kim, et al. | 1 | 0.95 | 0.85 | 1.07 | OR | HTN |  | mortality | Logistic | 13 |
| 33128848 | Kim, et al. | 1 | 1.19 | 0.97 | 1.45 | OR | CKD |  | mortality | Logistic | 13 |
| 33128848 | Kim, et al. | 1 | 1.55 | 1.40 | 1.71 | OR | Sex | Male | mortality | Logistic | 13 |
| 33128848 | Kim, et al. | 1 | 5.91 | 5.03 | 6.95 | OR | Age | >=80 | mortality | Logistic | 13 |
| 33128848 | Kim, et al. | 1 | 1.40 | 1.11 | 1.76 | OR | ESRD |  | mortality | Logistic | 13 |
| 33128848 | Kim, et al. | 1 | 1.33 | 1.11 | 1.60 | OR | COPD |  | mortality | Logistic | 13 |
| 33128848 | Kim, et al. | 1 | 1.25 | 1.03 | 1.52 | OR | BMI | 35-39.9 | mortality | Logistic | 13 |
| 33128848 | Kim, et al. | 1 | 1.04 | 0.92 | 1.17 | OR | BMI | 25-29.9 | mortality | Logistic | 13 |
| 33130213 | Tehrani, et al. | 1 | 1.01 | 0.42 | 2.42 | OR | Stroke |  | mortality | Logistic | 4 |
| 33130213 | Tehrani, et al. | 1 | 2.82 | 1.06 | 7.51 | OR | Heart disease |  | mortality | Logistic | 4 |
| 33130213 | Tehrani, et al. | 1 | 0.92 | 0.45 | 1.92 | OR | CKD |  | mortality | Logistic | 4 |
| 33130213 | Tehrani, et al. | 1 | 3.30 | 1.54 | 7.10 | OR | HF |  | mortality | Logistic | 4 |
| 33134966 | Zali, et al. | 1 | 2.21 | 1.99 | 2.45 | HR | Age | >=65 | mortality | Cox | 2 |
| 33134966 | Zali, et al. | 1 | 1.22 | 1.11 | 1.35 | HR | Sex | male | mortality | Cox | 2 |
| 33141353 | Farrell, et al. | 1 | 5.07 | 0.68 | 38.00 | HR | Comorbidity |  | mortality | Cox | 1 |
| 33141353 | Farrell, et al. | 1 | 1.04 | 1.01 | 1.06 | HR | Age | years | mortality | Cox | 1 |
| 33141353 | Farrell, et al. | 1 | 1.59 | 0.78 | 3.27 | HR | Sex | male | mortality | Cox | 1 |
| 33169090 | Shah, et al. | 1 | 3.33 | 1.07 | 10.41 | OR | cardiomyopathy |  | mortality | Logistic | 11 |
| 33169090 | Shah, et al. | 1 | 1.44 | 0.77 | 2.71 | OR | Cancer |  | mortality | Logistic | 11 |
| 33169090 | Shah, et al. | 1 | 1.21 | 0.70 | 2.09 | OR | COPD |  | mortality | Logistic | 11 |
| 33169090 | Shah, et al. | 1 | 1.54 | 0.73 | 3.26 | OR | Stroke |  | mortality | Logistic | 11 |
| 33169090 | Shah, et al. | 1 | 1.04 | 0.59 | 1.83 | OR | HTN |  | mortality | Logistic | 11 |
| 33169090 | Shah, et al. | 1 | 3.87 | 2.01 | 7.46 | OR | Age | >=65 | mortality | Logistic | 11 |
| 33169090 | Shah, et al. | 1 | 1.09 | 0.67 | 1.78 | OR | Diabetes |  | mortality | Logistic | 11 |
| 33169090 | Shah, et al. | 1 | 1.38 | 0.76 | 2.50 | OR | Dementia |  | mortality | Logistic | 11 |
| 33169090 | Shah, et al. | 1 | 0.91 | 0.50 | 1.65 | OR | AF |  | mortality | Logistic | 11 |
| 33169090 | Shah, et al. | 1 | 1.36 | 0.83 | 2.21 | OR | hyperlipidemia |  | mortality | Logistic | 11 |
| 33169090 | Shah, et al. | 1 | 1.24 | 0.78 | 1.97 | OR | Sex | male | mortality | Logistic | 11 |
| 33172229 | Ayaz, et al. | 1 | 20.50 | 2.40 | 177.30 | OR | Age | >=60 | mortality | Logistic | 4 |
| 33172229 | Ayaz, et al. | 1 | 26.50 | 4.70 | 147.80 | OR | Heart disease |  | mortality | Logistic | 4 |
| 33172229 | Ayaz, et al. | 1 | 18.80 | 2.20 | 162.40 | OR | Diabetes |  | mortality | Logistic | 4 |
| 33172229 | Ayaz, et al. | 1 | 12.70 | 1.50 | 108.80 | OR | HTN |  | mortality | Logistic | 4 |
| 33173851 | Rodriguez-Nava, et al. | 1 | 1.20 | 0.75 | 2.09 | HR | HTN |  | mortality | Cox | 4 |
| 33173851 | Rodriguez-Nava, et al. | 1 | 1.48 | 0.90 | 2.43 | HR | neurocog impairment |  | mortality | Cox | 4 |
| 33173851 | Rodriguez-Nava, et al. | 1 | 1.01 | 0.98 | 1.04 | HR | BMI |  | mortality | Cox | 4 |
| 33173851 | Rodriguez-Nava, et al. | 1 | 1.02 | 1.01 | 1.04 | HR | Age |  | mortality | Cox | 4 |
| 33218161 | Lee, et al. | 1 | 2.17 | 1.55 | 3.03 | OR | Diabetes |  | mortality | Logistic | 10 |
| 33218161 | Lee, et al. | 1 | 1.04 | 0.56 | 1.93 | OR | Cancer |  | mortality | Logistic | 10 |
| 33218161 | Lee, et al. | 1 | 2.10 | 1.51 | 2.92 | OR | Sex | male | mortality | Logistic | 10 |
| 33218161 | Lee, et al. | 1 | 3.11 | 1.33 | 7.30 | OR | CKD |  | mortality | Logistic | 10 |
| 33218161 | Lee, et al. | 1 | 0.68 | 0.38 | 1.21 | OR | Asthma |  | mortality | Logistic | 10 |
| 33218161 | Lee, et al. | 1 | 0.85 | 0.61 | 1.18 | OR | HTN |  | mortality | Logistic | 10 |
| 33218161 | Lee, et al. | 1 | 1.39 | 0.65 | 2.97 | OR | COPD |  | mortality | Logistic | 10 |
| 33218161 | Lee, et al. | 1 | 0.87 | 0.50 | 1.51 | OR | CLD |  | mortality | Logistic | 10 |
| 33218161 | Lee, et al. | 1 | 1.12 | 1.11 | 1.14 | OR | Age |  | mortality | Logistic | 10 |
| 33218161 | Lee, et al. | 1 | 0.82 | 0.56 | 1.22 | OR | CVD |  | mortality | Logistic | 10 |
| 33229434 | Loffi, et al. | 1 | 1.10 | 0.80 | 1.50 | HR | Diabetes |  | mortality | Cox | 10 |
| 33229434 | Loffi, et al. | 1 | 1.23 | 0.84 | 1.80 | HR | Smoking |  | mortality | Cox | 10 |
| 33229434 | Loffi, et al. | 1 | 1.06 | 0.72 | 1.57 | HR | cerebrovasc disease |  | mortality | Cox | 10 |
| 33229434 | Loffi, et al. | 1 | 0.63 | 0.49 | 0.82 | HR | Sex | female | mortality | Cox | 10 |
| 33229434 | Loffi, et al. | 1 | 1.62 | 1.53 | 1.72 | HR | Age | per 5 year increase | mortality | Cox | 10 |
| 33229434 | Loffi, et al. | 1 | 1.13 | 0.88 | 1.46 | HR | HTN |  | mortality | Cox | 10 |
| 33229434 | Loffi, et al. | 1 | 1.14 | 0.79 | 1.63 | HR | CAD |  | mortality | Cox | 10 |
| 33229434 | Loffi, et al. | 1 | 1.33 | 0.92 | 1.93 | HR | CKD |  | mortality | Cox | 10 |
| 33229434 | Loffi, et al. | 1 | 1.09 | 0.75 | 1.58 | HR | AF |  | mortality | Cox | 10 |
| 33229434 | Loffi, et al. | 1 | 0.94 | 0.63 | 1.41 | HR | hyperlipidemia |  | mortality | Cox | 10 |
| 33246431 | van Halem, et al. | 1 | 1.07 | 1.04 | 1.10 | OR | Age | years | mortality | Logistic | 4 |
| 33272355 | Kaeuffer, et al. | 1 | 1.70 | 1.10 | 2.70 | OR | Sex | male | mortality | Logistic | 8 |
| 33272355 | Kaeuffer, et al. | 1 | 2.30 | 1.30 | 3.90 | OR | CKD |  | mortality | Logistic | 8 |
| 33272355 | Kaeuffer, et al. | 1 | 1.70 | 1.00 | 2.70 | OR | Diabetes |  | mortality | Logistic | 8 |
| 33272355 | Kaeuffer, et al. | 1 | 0.60 | 0.30 | 0.90 | OR | HTN |  | mortality | Logistic | 8 |
| 33272355 | Kaeuffer, et al. | 1 | 2.70 | 2.10 | 3.50 | OR | Age | per 10 yr increase | mortality | Logistic | 8 |
| 33272355 | Kaeuffer, et al. | 1 | 0.90 | 0.50 | 1.50 | OR | Lung Disease |  | mortality | Logistic | 8 |
| 33272355 | Kaeuffer, et al. | 1 | 1.40 | 0.70 | 2.50 | OR | BMI | >=30 | mortality | Logistic | 8 |
| 33272355 | Kaeuffer, et al. | 1 | 0.90 | 0.50 | 1.60 | OR | BMI | 25-29.9 | mortality | Logistic | 8 |
| 33272355 | Kaeuffer, et al. | 1 | 3.80 | 1.60 | 7.70 | OR | Immunosuppressed |  | mortality | Logistic | 8 |
| 33278893 | Alguwaihes, et al. | 1 | 1.00 | 0.60 | 1.60 | HR | BMI | "obesity" | mortality | Cox | 10 |
| 33278893 | Alguwaihes, et al. | 1 | 1.30 | 0.50 | 3.80 | HR | Stroke |  | mortality | Cox | 10 |
| 33278893 | Alguwaihes, et al. | 1 | 0.70 | 0.20 | 2.00 | HR | CKD |  | mortality | Cox | 10 |
| 33278893 | Alguwaihes, et al. | 1 | 0.80 | 0.40 | 1.60 | HR | HTN |  | mortality | Cox | 10 |
| 33278893 | Alguwaihes, et al. | 1 | 1.20 | 0.70 | 2.30 | HR | Diabetes |  | mortality | Cox | 10 |
| 33278893 | Alguwaihes, et al. | 1 | 5.80 | 2.00 | 17.20 | HR | Smoking |  | mortality | Cox | 10 |
| 33278893 | Alguwaihes, et al. | 1 | 3.00 | 1.70 | 5.30 | HR | Age | >55 | mortality | Cox | 10 |
| 33278893 | Alguwaihes, et al. | 1 | 1.40 | 0.90 | 2.40 | HR | Sex | male | mortality | Cox | 10 |
| 33278893 | Alguwaihes, et al. | 1 | 1.80 | 0.70 | 4.40 | HR | CVD |  | mortality | Cox | 10 |
| 33278893 | Alguwaihes, et al. | 1 | 3.50 | 1.40 | 8.30 | HR | CHF |  | mortality | Cox | 10 |
| 33298991 | Rechtman, et al. | 1 | 1.03 | 1.02 | 1.04 | OR | BMI | per unit | mortality | Logistic | 3 |
| 33298991 | Rechtman, et al. | 1 | 1.79 | 1.67 | 1.93 | OR | Age | per decade | mortality | Logistic | 3 |
| 33298991 | Rechtman, et al. | 1 | 1.58 | 1.31 | 1.91 | OR | Sex | male | mortality | Logistic | 3 |
| 33324068 | Omar, et al. | 1 | 1.05 | 1.01 | 1.10 | OR | Age |  | mortality | Logistic | 2 |
| 33324068 | Omar, et al. | 1 | 0.73 | 0.96 | 14.22 | OR | Diabetes |  | mortality | Logistic | 2 |
| 33331576 | Caliskan, et al. | 0 | 1.76 | 0.41 | 7.61 | OR | Dementia |  | Mortality | Logistic | 9 |
| 33331576 | Caliskan, et al. | 0 | 6.25 | 2.17 | 18.00 | OR | CAD |  | Mortality | Logistic | 9 |
| 33331576 | Caliskan, et al. | 0 | 1.46 | 0.69 | 3.08 | OR | HTN |  | Mortality | Logistic | 9 |
| 33331576 | Caliskan, et al. | 0 | 3.21 | 1.22 | 8.43 | OR | COPD |  | Mortality | Logistic | 9 |
| 33331576 | Caliskan, et al. | 0 | 1.04 | 0.45 | 2.42 | OR | Diabetes |  | Mortality | Logistic | 9 |
| 33331576 | Caliskan, et al. | 0 | 1.08 | 1.06 | 1.11 | OR | Age |  | Mortality | Logistic | 9 |
| 33331576 | Caliskan, et al. | 0 | 6.51 | 2.73 | 15.50 | OR | Smoking | former | Mortality | Logistic | 9 |
| 33331576 | Caliskan, et al. | 0 | 13.01 | 5.06 | 33.48 | OR | Smoking | current | Mortality | Logistic | 9 |
| 33331576 | Caliskan, et al. | 0 | 5.92 | 1.07 | 32.26 | OR | CHF |  | Mortality | Logistic | 9 |
| 33331576 | Caliskan, et al. | 0 | 3.98 | 0.94 | 16.89 | OR | CKD |  | Mortality | Logistic | 9 |
| 33333477 | Moradi, et al. | 0 | 1.31 | 0.61 | 2.80 | HR | Sex | male | mortality | Cox | 0 |
| 33333477 | Moradi, et al. | 0 | 1.32 | 0.65 | 2.70 | HR | HTN |  | mortality | Cox | 0 |
| 33333477 | Moradi, et al. | 0 | 3.02 | 1.47 | 6.16 | HR | Heart disease |  | mortality | Cox | 0 |
| 33333477 | Moradi, et al. | 0 | 1.05 | 1.02 | 1.07 | HR | Age |  | mortality | Cox | 0 |
| 33333477 | Moradi, et al. | 0 | 1.26 | 0.44 | 3.60 | HR | Smoking |  | mortality | Cox | 0 |
| 33333477 | Moradi, et al. | 0 | 0.90 | 0.40 | 1.80 | HR | Diabetes |  | mortality | Cox | 0 |
| 33334400 | Redondo-Bravo, et al. | 1 | 1.47 | 1.29 | 1.68 | OR | CKD |  | hospitalization + Mortality | Logistic | 7 |
| 33334400 | Redondo-Bravo, et al. | 1 | 1.32 | 1.23 | 1.42 | OR | CVD |  | hospitalization + Mortality | Logistic | 7 |
| 33334400 | Redondo-Bravo, et al. | 1 | 1.33 | 1.24 | 1.42 | OR | Sex | Male | hospitalization + Mortality | Logistic | 7 |
| 33334400 | Redondo-Bravo, et al. | 1 | 4.84 | 3.37 | 6.96 | OR | Age | 60-69 | hospitalization + Mortality | Logistic | 7 |
| 33334400 | Redondo-Bravo, et al. | 1 | 10.01 | 6.99 | 14.35 | OR | Age | 70-79 | hospitalization + Mortality | Logistic | 7 |
| 33334400 | Redondo-Bravo, et al. | 1 | 1.14 | 1.04 | 1.24 | OR | Lung Disease |  | hospitalization + Mortality | Logistic | 7 |
| 33334400 | Redondo-Bravo, et al. | 1 | 0.90 | 0.82 | 0.98 | OR | HTN |  | hospitalization + Mortality | Logistic | 7 |
| 33334400 | Redondo-Bravo, et al. | 1 | 28.45 | 19.85 | 40.78 | OR | Age | >=80 | hospitalization + Mortality | Logistic | 7 |
| 33334400 | Redondo-Bravo, et al. | 1 | 1.91 | 1.32 | 2.77 | OR | Age | 40-59 | hospitalization + Mortality | Logistic | 7 |
| 33334400 | Redondo-Bravo, et al. | 1 | 1.23 | 1.14 | 1.33 | OR | Diabetes |  | hospitalization + Mortality | Logistic | 7 |
| 33334842 | Elimian, et al. | 1 | 1.39 | 0.51 | 3.75 | OR | Age | 41-50 | mortality | Logistic | 2 |
| 33334842 | Elimian, et al. | 1 | 2.93 | 1.10 | 7.81 | OR | Age | 51-60 | mortality | Logistic | 2 |
| 33334842 | Elimian, et al. | 1 | 0.57 | 0.19 | 1.68 | OR | Age | 21-30 | mortality | Logistic | 2 |
| 33334842 | Elimian, et al. | 1 | 1.23 | 0.76 | 2.00 | OR | Sex | male | mortality | Logistic | 2 |
| 33334842 | Elimian, et al. | 1 | 10.81 | 3.70 | 31.60 | OR | Age | >=71 | mortality | Logistic | 2 |
| 33334842 | Elimian, et al. | 1 | 0.64 | 0.22 | 1.80 | OR | Age | 31-40 | mortality | Logistic | 2 |
| 33334842 | Elimian, et al. | 1 | 14.52 | 5.35 | 39.43 | OR | Age | 61-70 | mortality | Logistic | 2 |
| 33338063 | Matangila, et al. | 0 | 1.88 | 0.76 | 4.65 | OR | Diabetes |  | mortality | Logistic | 0 |
| 33338063 | Matangila, et al. | 0 | 1.30 | 0.33 | 5.11 | OR | BMI | "obesity" | mortality | Logistic | 0 |
| 33338063 | Matangila, et al. | 0 | 1.04 | 1.01 | 0.07 | OR | Age |  | mortality | Logistic | 0 |
| 33338063 | Matangila, et al. | 0 | 1.59 | 0.72 | 3.53 | OR | HTN |  | mortality | Logistic | 0 |
| 33338063 | Matangila, et al. | 0 | 2.35 | 0.64 | 8.58 | OR | Heart disease |  | mortality | Logistic | 0 |
| 33338063 | Matangila, et al. | 0 | 1.06 | 0.49 | 2.32 | OR | Sex | male | mortality | Logistic | 0 |
| 33354690 | Chishinga, et al. | 1 | 1.70 | 1.20 | 2.40 | OR | Sex | male | hospitalization + mortality | Logistic | 9 |
| 33354690 | Chishinga, et al. | 1 | 1.00 | 0.60 | 1.60 | OR | Diabetes |  | hospitalization + mortality | Logistic | 9 |
| 33354690 | Chishinga, et al. | 1 | 0.40 | 0.00 | 2.80 | OR | Age | 35-44 | hospitalization + mortality | Logistic | 9 |
| 33354690 | Chishinga, et al. | 1 | 1.50 | 0.80 | 3.10 | OR | Immunosuppressed |  | hospitalization + mortality | Logistic | 9 |
| 33354690 | Chishinga, et al. | 1 | 24.10 | 5.20 | 111.70 | OR | Age | >=75 | hospitalization + mortality | Logistic | 9 |
| 33354690 | Chishinga, et al. | 1 | 1.90 | 0.40 | 8.20 | OR | CLD |  | hospitalization + mortality | Logistic | 9 |
| 33354690 | Chishinga, et al. | 1 | 3.00 | 1.70 | 5.30 | OR | Neurologic disease |  | hospitalization + mortality | Logistic | 9 |
| 33354690 | Chishinga, et al. | 1 | 0.70 | 0.10 | 4.80 | OR | Age | 25-34 | hospitalization + mortality | Logistic | 9 |
| 33354690 | Chishinga, et al. | 1 | 9.00 | 1.90 | 41.40 | OR | Age | 65-74 | hospitalization + mortality | Logistic | 9 |
| 33354690 | Chishinga, et al. | 1 | 1.10 | 0.60 | 1.90 | OR | Lung Disease |  | hospitalization + mortality | Logistic | 9 |
| 33354690 | Chishinga, et al. | 1 | 1.90 | 1.10 | 3.20 | OR | CVD |  | hospitalization + mortality | Logistic | 9 |
| 33354690 | Chishinga, et al. | 1 | 3.80 | 0.80 | 17.60 | OR | Age | 55-64 | hospitalization + mortality | Logistic | 9 |
| 33354690 | Chishinga, et al. | 1 | 2.30 | 0.50 | 11.20 | OR | Age | 45-54 | hospitalization + mortality | Logistic | 9 |
| 33354690 | Chishinga, et al. | 1 | 1.70 | 0.90 | 3.10 | OR | CKD |  | hospitalization + mortality | Logistic | 9 |
| 33370364 | Mejia, et al. | 1 | 0.81 | 0.58 | 1.13 | HR | Sex | male | mortality | Cox | 5 |
| 33370364 | Mejia, et al. | 1 | 1.20 | 0.85 | 1.70 | HR | HTN |  | mortality | Cox | 5 |
| 33370364 | Mejia, et al. | 1 | 1.88 | 1.32 | 2.69 | HR | Age | >=60 | mortality | Cox | 5 |
| 33370364 | Mejia, et al. | 1 | 1.10 | 0.77 | 1.59 | HR | Diabetes |  | mortality | Cox | 5 |
| 33370364 | Mejia, et al. | 1 | 0.99 | 0.72 | 1.35 | HR | BMI | "obesity" | mortality | Cox | 5 |
| 33390322 | McNeill, et al. | 1 | 2.20 | 1.21 | 3.70 | OR | BMI | >=30 | mortality | Logistic | 8 |
| 33393318 | Javanian, et al. | 1 | 1.09 | 0.73 | 1.62 | RR | Sex | male | mortality | Poisson | 4 |
| 33393318 | Javanian, et al. | 1 | 1.96 | 1.20 | 3.19 | RR | cerebrovasc disease |  | mortality | Poisson | 4 |
| 33393318 | Javanian, et al. | 1 | 1.03 | 1.01 | 1.05 | RR | Age |  | mortality | Poisson | 4 |
| 33393318 | Javanian, et al. | 1 | 1.01 | 0.68 | 1.47 | RR | HTN |  | mortality | Poisson | 4 |
| 33395425 | Ortiz-Prado, et al. | 1 | 2.03 | 1.62 | 2.50 | OR | Sex | male | mortality | Logistic | 3 |
| 33395425 | Ortiz-Prado, et al. | 1 | 5.74 | 4.71 | 7.00 | OR | Age | >65 | mortality | Logistic | 3 |
| 33395425 | Ortiz-Prado, et al. | 1 | 2.27 | 1.72 | 3.00 | OR | Comorbidity | present | mortality | Logistic | 3 |
| 33409033 | Rustgi, et al. | 1 | 2.40 | 1.14 | 5.04 | OR | CKD |  | mortality | Logistic | 2 |
| 33409033 | Rustgi, et al. | 1 | 1.04 | 1.03 | 1.06 | OR | Age | years | mortality | Logistic | 2 |
| 33410296 | Kristic, et al. | 1 | 19.60 | 13.60 | 28.40 | OR | Age | 65-79 | mortality | Logistic | 4 |
| 33410296 | Kristic, et al. | 1 | 58.20 | 39.50 | 85.80 | OR | Age | 80+ | mortality | Logistic | 4 |
| 33410296 | Kristic, et al. | 1 | 0.51 | 0.39 | 0.66 | OR | Sex | female | mortality | Logistic | 4 |
| 33444432 | Saurabh, et al. | 0 | 5.55 | 1.84 | 16.69 | OR | Age | 60-69 | mortality | Logistic | 0 |
| 33444432 | Saurabh, et al. | 0 | 4.91 | 1.51 | 15.95 | OR | Age | >=70 | mortality | Logistic | 0 |
| 33444432 | Saurabh, et al. | 0 | 1.05 | 0.54 | 2.04 | OR | Sex | male | mortality | Logistic | 0 |
| 33444432 | Saurabh, et al. | 0 | 1.38 | 0.38 | 4.98 | OR | Age | 50-59 | mortality | Logistic | 0 |
| 33449333 | Salari, et al. | 1 | 2.49 | 1.15 | 5.41 | OR | BMI | >=40 | mortality | Logistic | 3 |
| 33468081 | Rastad, et al | 1 | 2.59 | 1.55 | 4.32 | HR | ESRD |  | mortality | Cox | 2 |
| 33500292 | Mash, et al. | 1 | 2.02 | 1.37 | 2.98 | OR | Sex |  | mortality | Logistic | 8 |
| 33500292 | Mash, et al. | 1 | 1.06 | 1.04 | 1.07 | OR | Age |  | mortality | Logistic | 8 |
| 33500292 | Mash, et al. | 1 | 3.41 | 2.06 | 5.65 | OR | HIV |  | mortality | Logistic | 8 |
| 33500292 | Mash, et al. | 1 | 1.84 | 1.24 | 2.73 | OR | Diabetes |  | mortality | Logistic | 8 |
| 33500292 | Mash, et al. | 1 | 7.45 | 1.87 | 29.89 | OR | Cancer |  | mortality | Logistic | 8 |
| 33500292 | Mash, et al. | 1 | 1.58 | 1.02 | 2.46 | OR | Obesity |  | mortality | Logistic | 8 |
| 33500292 | Mash, et al. | 1 | 5.16 | 2.82 | 9.43 | OR | CKD |  | mortality | Logistic | 8 |
| 33500292 | Mash, et al. | 1 | 2.85 | 1.52 | 5.35 | OR | cardiac failure |  | mortality | Logistic | 8 |
| 33519709 | Crouse, et al. | 1 | 1.21 | 0.66 | 2.21 | OR | BMI | >=30 | mortality | Logistic | 5 |
| 33519709 | Crouse, et al. | 1 | 3.03 | 1.67 | 5.52 | OR | Age | >70 | mortality | Logistic | 5 |
| 33519709 | Crouse, et al. | 1 | 1.00 | 0.42 | 2.38 | OR | HTN |  | mortality | Logistic | 5 |
| 33519709 | Crouse, et al. | 1 | 4.38 | 1.60 | 12.00 | OR | Age | 50-70 | mortality | Logistic | 5 |
| 33519709 | Crouse, et al. | 1 | 1.79 | 1.02 | 3.13 | OR | Sex | male | mortality | Logistic | 5 |
| 33519709 | Crouse, et al. | 1 | 2.40 | 1.29 | 4.48 | OR | Diabetes |  | mortality | Logistic | 5 |
| 33528146 | Kvale, et al. | 1 | 0.90 | 0.70 | 1.20 | RR | HTN |  | mortality | RRs | 4 |
| 33528146 | Kvale, et al. | 1 | 1.50 | 1.00 | 2.10 | RR | Stroke |  | mortality | RRs | 4 |
| 33528146 | Kvale, et al. | 1 | 306.40 | 163.00 | 575.80 | RR | Age | >=90 | mortality | RRs | 4 |
| 33528146 | Kvale, et al. | 1 | 1.50 | 1.20 | 2.00 | RR | Sex | male | mortality | RRs | 4 |
| 33528146 | Kvale, et al. | 1 | 3.00 | 1.10 | 8.20 | RR | metastatic cancer |  | mortality | RRs | 4 |
| 33528146 | Kvale, et al. | 1 | 178.40 | 93.80 | 339.50 | RR | Age | 85-90 | mortality | RRs | 4 |
| 33528146 | Kvale, et al. | 1 | 13.10 | 6.50 | 26.60 | RR | Age | 60-69 | mortality | RRs | 4 |
| 33528146 | Kvale, et al. | 1 | 0.80 | 0.50 | 1.20 | RR | regional cancer |  | mortality | RRs | 4 |
| 33528146 | Kvale, et al. | 1 | 1.20 | 0.80 | 1.60 | RR | AF |  | mortality | RRs | 4 |
| 33528146 | Kvale, et al. | 1 | 51.00 | 27.20 | 95.70 | RR | Age | 70-79 | mortality | RRs | 4 |
| 33528146 | Kvale, et al. | 1 | 105.40 | 54.90 | 202.10 | RR | Age | 80-84 | mortality | RRs | 4 |
| 33528146 | Kvale, et al. | 1 | 0.80 | 0.40 | 1.30 | RR | MI |  | mortality | RRs | 4 |
| 33528146 | Kvale, et al. | 1 | 0.90 | 0.60 | 1.40 | RR | HF |  | mortality | RRs | 4 |

**pmid, PubMed ID**

**adj, adjusted (0=no, 1=yes)**

**lb, lower bound**

**ub, upper bound**

**est_meth, estimation method**

**cov_num, number of covariates adjusted for**
