# Supplementary material for: Risk factors for SARS-CoV-2 related mortality and hospitalization before vaccination: A meta-analysis
Source: PLOS Glob Public Health. 2022 Nov 2;2(11):e0001187. doi: 10.1371/journal.pgph.0001187 (PMC10021978; doi:10.1371/journal.pgph.0001187)
Supplement: S4 Table — (DOCX) [file pgph.0001187.s013.docx]

**S4 Table. Raw data extracted from individual hospitalization studies**

| **pmid** | **adj** | **estimate** | **lb** | **ub** | **measure** | **risk factor** | **category** | **lastname** | **firstname** | **journal** | **cov_num** | **outcome** | **est_meth** |
| --- | --- | --- | --- | --- | --- | --- | --- | --- | --- | --- | --- | --- | --- |
| 32437224 | 1 | 0.26 | 0.08 | 0.80 | OR | Smoking | unknown | Azar | Kristen | Health Affairs | 15 | hospitalization | 2 |
| 32437224 | 1 | 1.05 | 0.59 | 1.84 | OR | insurance | medicare | Azar | Kristen | Health Affairs | 15 | hospitalization | 2 |
| 32437224 | 1 | 2.17 | 1.33 | 3.53 | OR | Diabetes |  | Azar | Kristen | Health Affairs | 15 | hospitalization | 2 |
| 32437224 | 1 | 3.34 | 1.49 | 7.52 | OR | CHF |  | Azar | Kristen | Health Affairs | 15 | hospitalization | 2 |
| 32437224 | 1 | 1.09 | 0.37 | 3.19 | OR | Smoking | never | Azar | Kristen | Health Affairs | 15 | hospitalization | 2 |
| 32437224 | 1 | 1.40 | 0.93 | 2.10 | OR | HTN |  | Azar | Kristen | Health Affairs | 15 | hospitalization | 2 |
| 32437224 | 1 | 1.80 | 0.75 | 4.34 | OR | asthma or COPD |  | Azar | Kristen | Health Affairs | 15 | hospitalization | 2 |
| 32437224 | 1 | 4.43 | 0.35 | 56.48 | OR | insurance | other | Azar | Kristen | Health Affairs | 15 | hospitalization | 2 |
| 32437224 | 1 | 5.68 | 2.60 | 12.38 | OR | Age |  | Azar | Kristen | Health Affairs | 15 | hospitalization | 2 |
| 32437224 | 1 | 1.32 | 0.75 | 2.32 | OR | CVD |  | Azar | Kristen | Health Affairs | 15 | hospitalization | 2 |
| 32437224 | 1 | 2.24 | 1.13 | 4.43 | OR | Age |  | Azar | Kristen | Health Affairs | 15 | hospitalization | 2 |
| 32437224 | 1 | 2.13 | 1.24 | 3.68 | OR | insurance | medicaid | Azar | Kristen | Health Affairs | 15 | hospitalization | 2 |
| 32437224 | 1 | 0.96 | 0.45 | 2.03 | OR | cancer |  | Azar | Kristen | Health Affairs | 15 | hospitalization | 2 |
| 32437224 | 1 | 0.77 | 0.25 | 2.35 | OR | Smoking | past | Azar | Kristen | Health Affairs | 15 | hospitalization | 2 |
| 32437224 | 1 | 4.62 | 2.39 | 9.95 | OR | Age |  | Azar | Kristen | Health Affairs | 15 | hospitalization | 2 |
| 32437224 | 1 | 2.19 | 1.03 | 4.36 | OR | insurance | self | Azar | Kristen | Health Affairs | 15 | hospitalization | 2 |
| 32437224 | 1 | 2.62 | 1.37 | 4.99 | OR | Age |  | Azar | Kristen | Health Affairs | 15 | hospitalization | 2 |
| 32437224 | 1 | 1.52 | 0.89 | 2.58 | OR | asthma or COPD |  | Azar | Kristen | Health Affairs | 15 | hospitalization | 2 |
| 32437224 | 1 | 19.08 | 7.86 | 46.32 | OR | Age |  | Azar | Kristen | Health Affairs | 15 | hospitalization | 2 |
| 32437224 | 1 | 1.94 | 1.33 | 2.81 | OR | Sex | male | Azar | Kristen | Health Affairs | 15 | hospitalization | 2 |
| 32444366 | 1 | 0.59 | 0.43 | 0.81 | OR | Smoking | current | Petrilli | Christopher | BMJ | 14 | hospitalization | 2 |
| 32444366 | 1 | 4.43 | 2.59 | 8.04 | OR | CHF |  | Petrilli | Christopher | BMJ | 14 | hospitalization | 2 |
| 32444366 | 1 | 0.88 | 0.65 | 1.19 | OR | cancer |  | Petrilli | Christopher | BMJ | 14 | hospitalization | 2 |
| 32444366 | 1 | 2.14 | 1.76 | 2.59 | OR | Age |  | Petrilli | Christopher | BMJ | 14 | hospitalization | 2 |
| 32444366 | 1 | 2.76 | 2.39 | 3.20 | OR | Sex | male | Petrilli | Christopher | BMJ | 14 | hospitalization | 2 |
| 32444366 | 1 | 1.43 | 1.16 | 1.75 | OR | Smoking | unknown | Petrilli | Christopher | BMJ | 14 | hospitalization | 2 |
| 32444366 | 1 | 0.69 | 0.56 | 0.85 | OR | Smoking | past | Petrilli | Christopher | BMJ | 14 | hospitalization | 2 |
| 32444366 | 1 | 1.08 | 0.81 | 1.44 | OR | CAD |  | Petrilli | Christopher | BMJ | 14 | hospitalization | 2 |
| 32444366 | 1 | 3.67 | 3.01 | 4.48 | OR | Age |  | Petrilli | Christopher | BMJ | 14 | hospitalization | 2 |
| 32444366 | 1 | 2.60 | 1.89 | 3.61 | OR | CKD |  | Petrilli | Christopher | BMJ | 14 | hospitalization | 2 |
| 32444366 | 1 | 8.70 | 6.77 | 11.22 | OR | Age |  | Petrilli | Christopher | BMJ | 14 | hospitalization | 2 |
| 32444366 | 1 | 2.24 | 1.84 | 2.73 | OR | Diabetes |  | Petrilli | Christopher | BMJ | 14 | hospitalization | 2 |
| 32444366 | 1 | 1.08 | 0.88 | 1.33 | OR | asthma or COPD |  | Petrilli | Christopher | BMJ | 14 | hospitalization | 2 |
| 32444366 | 1 | 1.78 | 1.49 | 2.12 | OR | HTN |  | Petrilli | Christopher | BMJ | 14 | hospitalization | 2 |
| 32444366 | 1 | 37.87 | 26.10 | 56.03 | OR | Age |  | Petrilli | Christopher | BMJ | 14 | hospitalization | 2 |
| 32459916 | 0 | 1.73 | 1.39 | 2.14 | OR | insurance | medicare | Price-Haywood | Eboni | NEJM |  | hospitalization | 2 |
| 32459916 | 0 | 0.91 | 0.70 | 1.20 | OR | insurance | other | Price-Haywood | Eboni | NEJM |  | hospitalization | 2 |
| 32459916 | 0 | 0.56 | 0.48 | 0.65 | OR | Sex |  | Price-Haywood | Eboni | NEJM |  | hospitalization | 2 |
| 32459916 | 0 | 1.65 | 1.29 | 2.12 | OR | insurance | medicaid | Price-Haywood | Eboni | NEJM |  | hospitalization | 2 |
| 32459916 | 0 | 1.43 | 1.20 | 1.71 | OR | Obesity |  | Price-Haywood | Eboni | NEJM |  | hospitalization | 2 |
| 32459916 | 0 | 1.29 | 1.25 | 1.33 | OR | Age |  | Price-Haywood | Eboni | NEJM |  | hospitalization | 2 |
| 32497776 | 1 | 1.02 | 1.01 | 1.03 | OR | Age |  | Lassale | Camille | Brain Behav Immun | all covariates | hospitalization | 2 |
| 32497776 | 1 | 1.30 | 1.10 | 1.55 | OR | Smoking | past | Lassale | Camille | Brain Behav Immun | all covariates | hospitalization | 2 |
| 32497776 | 1 | 1.25 | 0.96 | 1.62 | OR | Smoking | current | Lassale | Camille | Brain Behav Immun | all covariates | hospitalization | 2 |
| 32497776 | 1 | 1.10 | 0.90 | 1.34 | OR | alcohol | above | Lassale | Camille | Brain Behav Immun | all covariates | hospitalization | 2 |
| 32497776 | 1 | 0.93 | 0.77 | 1.13 | OR | activity | insufficient | Lassale | Camille | Brain Behav Immun | all covariates | hospitalization | 2 |
| 32497776 | 1 | 0.98 | 0.82 | 1.17 | OR | HTN |  | Lassale | Camille | Brain Behav Immun | all covariates | hospitalization | 2 |
| 32497776 | 1 | 1.30 | 1.07 | 1.59 | OR | alcohol | rarely | Lassale | Camille | Brain Behav Immun | all covariates | hospitalization | 2 |
| 32497776 | 1 | 1.06 | 0.79 | 1.42 | OR | CVD |  | Lassale | Camille | Brain Behav Immun | all covariates | hospitalization | 2 |
| 32497776 | 1 | 1.22 | 1.00 | 1.48 | OR | activity | none | Lassale | Camille | Brain Behav Immun | all covariates | hospitalization | 2 |
| 32497776 | 1 | 1.15 | 0.92 | 1.44 | OR | Sex | male | Lassale | Camille | Brain Behav Immun | all covariates | hospitalization | 2 |
| 32497776 | 1 | 1.03 | 1.02 | 1.05 | OR | BMI |  | Lassale | Camille | Brain Behav Immun | all covariates | hospitalization | 2 |
| 32522462 | 1 | 1.39 | 1.13 | 1.71 | OR | asthma or COPD |  | Zhu | Zhaozhong | J Allergy Clin Immunol | 4 | hospitalization | 2 |
| 32607513 | 1 | 1.20 | 0.84 | 1.71 | OR | Sex | male | Mendy | Angelico | medRxiv | 4 | hospitalization | 2 |
| 32607513 | 1 | 3.47 | 1.99 | 6.07 | OR | CKD |  | Mendy | Angelico | medRxiv | 4 | hospitalization | 2 |
| 32607513 | 1 | 1.40 | 0.89 | 2.22 | OR | Obesity |  | Mendy | Angelico | medRxiv | 4 | hospitalization | 2 |
| 32607513 | 1 | 1.69 | 0.90 | 3.19 | OR | asthma or COPD |  | Mendy | Angelico | medRxiv | 4 | hospitalization | 2 |
| 32607513 | 1 | 1.36 | 1.22 | 1.51 | OR | Age |  | Mendy | Angelico | medRxiv | 4 | hospitalization | 2 |
| 32607513 | 1 | 1.92 | 1.10 | 3.35 | OR | asthma or COPD |  | Mendy | Angelico | medRxiv | 4 | hospitalization | 2 |
| 32607513 | 1 | 2.62 | 1.75 | 3.90 | OR | Diabetes |  | Mendy | Angelico | medRxiv | 4 | hospitalization | 2 |
| 32607513 | 1 | 4.39 | 2.75 | 7.01 | OR | CVD |  | Mendy | Angelico | medRxiv | 4 | hospitalization | 2 |
| 32607513 | 1 | 1.42 | 0.91 | 2.20 | OR | cancer |  | Mendy | Angelico | medRxiv | 4 | hospitalization | 2 |
| 32607513 | 1 | 2.01 | 1.32 | 3.06 | OR | Smoking | current | Mendy | Angelico | medRxiv | 4 | hospitalization | 2 |
| 32682453 | 1 | 1.34 | 1.10 | 1.16 | OR | Diabetes |  | Soares | Rita | Am J Trop Med Hyg | 15 | mortality | 2 |
| 32682453 | 1 | 3.40 | 2.91 | 3.96 | OR | Age |  | Soares | Rita | Am J Trop Med Hyg | 15 | mortality | 2 |
| 32682453 | 1 | 2.91 | 2.04 | 4.12 | OR | Smoking | current | Soares | Rita | Am J Trop Med Hyg | 15 | mortality | 2 |
| 32682453 | 1 | 1.43 | 1.25 | 1.65 | OR | Sex | male | Soares | Rita | Am J Trop Med Hyg | 15 | mortality | 2 |
| 32682453 | 1 | 1.74 | 1.35 | 2.23 | OR | Obesity |  | Soares | Rita | Am J Trop Med Hyg | 15 | mortality | 2 |
| 32682453 | 1 | 2.41 | 1.59 | 3.66 | OR | CKD |  | Soares | Rita | Am J Trop Med Hyg | 15 | mortality | 2 |
| 32682453 | 1 | 1.30 | 1.11 | 1.53 | OR | CVD |  | Soares | Rita | Am J Trop Med Hyg | 15 | mortality | 2 |
| 32700398 | 1 | 2.71 | 1.55 | 4.78 | OR | Age | >50 | Merzon | Eugene | FEBS J |  | hospitalization | 2 |
| 32700398 | 1 | 1.06 | 0.44 | 2.58 | OR | CVD |  | Merzon | Eugene | FEBS J |  | hospitalization | 2 |
| 32700398 | 1 | 1.35 | 0.83 | 2.21 | OR | Sex | male | Merzon | Eugene | FEBS J |  | hospitalization | 2 |
| 32700398 | 1 | 1.82 | 0.41 | 2.36 | OR | Diabetes |  | Merzon | Eugene | FEBS J |  | hospitalization | 2 |
| 32700398 | 1 | 0.94 | 0.52 | 1.71 | OR | Lung disease |  | Merzon | Eugene | FEBS J |  | hospitalization | 2 |
| 32700398 | 1 | 0.94 | 0.50 | 1.76 | OR | depression |  | Merzon | Eugene | FEBS J |  | hospitalization | 2 |
| 32700398 | 1 | 1.56 | 0.91 | 2.71 | OR | HTN |  | Merzon | Eugene | FEBS J |  | hospitalization | 2 |
| 32700398 | 1 | 1.22 | 0.71 | 2.08 | OR | Smoking |  | Merzon | Eugene | FEBS J |  | hospitalization | 2 |
| 32700398 | 1 | 1.52 | 0.46 | 4.98 | OR | Dementia |  | Merzon | Eugene | FEBS J |  | hospitalization | 2 |
| 32700398 | 1 | 0.99 | 0.98 | 1.01 | OR | BMI |  | Merzon | Eugene | FEBS J |  | hospitalization | 2 |
| 32722159 | 0 | 5.85 | 4.94 | 6.92 | OR | Cancer |  | Noguerira | Paulo Jorge | J Clin Med |  | hospitalization + mortality | 2 |
| 32722159 | 0 | 11.48 | 9.82 | 13.45 | OR | neuromuscular disorder |  | Noguerira | Paulo Jorge | J Clin Med |  | hospitalization + mortality | 2 |
| 32722159 | 0 | 0.59 | 0.38 | 0.87 | OR | Asthma |  | Noguerira | Paulo Jorge | J Clin Med |  | hospitalization + mortality | 2 |
| 32722159 | 0 | 5.40 | 4.75 | 6.14 | OR | Diabetes |  | Noguerira | Paulo Jorge | J Clin Med |  | hospitalization + mortality | 2 |
| 32722159 | 0 | 4.10 | 2.74 | 6.10 | OR | HIV |  | Noguerira | Paulo Jorge | J Clin Med |  | hospitalization + mortality | 2 |
| 32722159 | 0 | 4.85 | 4.12 | 5.69 | OR | Lung disease |  | Noguerira | Paulo Jorge | J Clin Med |  | hospitalization + mortality | 2 |
| 32722159 | 0 | 14.33 | 11.48 | 18.01 | OR | CKD |  | Noguerira | Paulo Jorge | J Clin Med |  | hospitalization + mortality | 2 |
| 32722159 | 0 | 10.15 | 7.61 | 13.64 | OR | Cancer |  | Noguerira | Paulo Jorge | J Clin Med |  | hospitalization + mortality | 2 |
| 32722159 | 0 | 87.66 | 32.19 | 361.00 | OR | Heart disease |  | Noguerira | Paulo Jorge | J Clin Med |  | hospitalization + mortality | 2 |
| 32722159 | 0 | 9.07 | 6.09 | 13.68 | OR | CLD |  | Noguerira | Paulo Jorge | J Clin Med |  | hospitalization + mortality | 2 |
| 32743602 | 0 | 1.07 | 0.58 | 1.92 | OR | lymphoma |  | McPadden | Jacob | medRxiv |  | hospitalization + mortality | 2 |
| 32743602 | 0 | 0.87 | 0.69 | 1.09 | OR | Cancer |  | McPadden | Jacob | medRxiv |  | hospitalization + mortality | 2 |
| 32743602 | 0 | 1.18 | 1.02 | 1.37 | OR | BMI | obesity | McPadden | Jacob | medRxiv |  | hospitalization + mortality | 2 |
| 32743602 | 0 | 1.06 | 0.83 | 1.36 | OR | CHF |  | McPadden | Jacob | medRxiv |  | hospitalization + mortality | 2 |
| 32743602 | 0 | 1.16 | 0.95 | 1.40 | OR | diabetes, uncomplicated |  | McPadden | Jacob | medRxiv |  | hospitalization + mortality | 2 |
| 32743602 | 0 | 1.31 | 1.07 | 1.61 | OR | Neurologic disease |  | McPadden | Jacob | medRxiv |  | hospitalization + mortality | 2 |
| 32743602 | 0 | 1.43 | 1.13 | 1.81 | OR | Age | 35-44 | McPadden | Jacob | medRxiv |  | hospitalization + mortality | 2 |
| 32743602 | 0 | 15.91 | 11.92 | 21.33 | OR | Age | 75-84 | McPadden | Jacob | medRxiv |  | hospitalization + mortality | 2 |
| 32743602 | 0 | 1.55 | 1.11 | 2.15 | OR | Cancer |  | McPadden | Jacob | medRxiv |  | hospitalization + mortality | 2 |
| 32743602 | 0 | 0.89 | 0.75 | 1.06 | OR | hypothyroidism |  | McPadden | Jacob | medRxiv |  | hospitalization + mortality | 2 |
| 32743602 | 0 | 22.03 | 16.10 | 30.30 | OR | Age | 85+ | McPadden | Jacob | medRxiv |  | hospitalization + mortality | 2 |
| 32743602 | 0 | 0.85 | 0.72 | 1.01 | OR | depression |  | McPadden | Jacob | medRxiv |  | hospitalization + mortality | 2 |
| 32743602 | 0 | 1.76 | 1.41 | 2.21 | OR | Age | 45-54 | McPadden | Jacob | medRxiv |  | hospitalization + mortality | 2 |
| 32743602 | 0 | 1.13 | 0.96 | 1.33 | OR | cardiac arrythmias |  | McPadden | Jacob | medRxiv |  | hospitalization + mortality | 2 |
| 32743602 | 0 | 6.95 | 5.45 | 8.91 | OR | Age | 65-74 | McPadden | Jacob | medRxiv |  | hospitalization + mortality | 2 |
| 32743602 | 0 | 0.89 | 0.69 | 1.15 | OR | RA/collagen vascular disease |  | McPadden | Jacob | medRxiv |  | hospitalization + mortality | 2 |
| 32743602 | 0 | 0.94 | 0.81 | 1.09 | OR | Lung disease |  | McPadden | Jacob | medRxiv |  | hospitalization + mortality | 2 |
| 32743602 | 0 | 1.01 | 0.83 | 1.23 | OR | valvular disease |  | McPadden | Jacob | medRxiv |  | hospitalization + mortality | 2 |
| 32743602 | 0 | 1.38 | 1.08 | 1.75 | OR | CKD |  | McPadden | Jacob | medRxiv |  | hospitalization + mortality | 2 |
| 32743602 | 0 | 1.14 | 0.88 | 1.48 | OR | HTN, complicated |  | McPadden | Jacob | medRxiv |  | hospitalization + mortality | 2 |
| 32743602 | 0 | 1.01 | 0.83 | 1.23 | OR | CLD |  | McPadden | Jacob | medRxiv |  | hospitalization + mortality | 2 |
| 32743602 | 0 | 1.18 | 0.94 | 1.47 | OR | diabetes, complicated |  | McPadden | Jacob | medRxiv |  | hospitalization + mortality | 2 |
| 32743602 | 0 | 0.97 | 0.83 | 1.13 | OR | HTN, uncomplicated |  | McPadden | Jacob | medRxiv |  | hospitalization + mortality | 2 |
| 32743602 | 0 | 1.26 | 0.71 | 2.20 | OR | HIV |  | McPadden | Jacob | medRxiv |  | hospitalization + mortality | 2 |
| 32743602 | 0 | 1.68 | 1.48 | 1.90 | OR | Sex | male | McPadden | Jacob | medRxiv |  | hospitalization + mortality | 2 |
| 32743602 | 0 | 3.24 | 2.60 | 4.04 | OR | Age | 55-64 | McPadden | Jacob | medRxiv |  | hospitalization + mortality | 2 |
| 32743602 | 0 | 0.84 | 0.68 | 1.04 | OR | PVD |  | McPadden | Jacob | medRxiv |  | hospitalization + mortality | 2 |
| 32747155 | 1 | 0.83 | 0.78 | 0.89 | OR | Asthma |  | Hernandez-Galdamez | Diego Rolando | Arch Med Res |  | hospitalization | 2 |
| 32747155 | 1 | 0.93 | 0.90 | 0.97 | OR | Smoking |  | Hernandez-Galdamez | Diego Rolando | Arch Med Res |  | hospitalization | 2 |
| 32747155 | 1 | 1.05 | 0.98 | 1.12 | OR | CVD |  | Hernandez-Galdamez | Diego Rolando | Arch Med Res |  | hospitalization | 2 |
| 32747155 | 1 | 1.34 | 1.24 | 1.44 | OR | COPD |  | Hernandez-Galdamez | Diego Rolando | Arch Med Res |  | hospitalization | 2 |
| 32747155 | 1 | 1.98 | 1.93 | 2.03 | OR | Diabetes |  | Hernandez-Galdamez | Diego Rolando | Arch Med Res |  | hospitalization | 2 |
| 32747155 | 1 | 1.29 | 1.25 | 1.32 | OR | BMI | obesity | Hernandez-Galdamez | Diego Rolando | Arch Med Res |  | hospitalization | 2 |
| 32747155 | 1 | 1.26 | 1.23 | 1.29 | OR | HTN |  | Hernandez-Galdamez | Diego Rolando | Arch Med Res |  | hospitalization | 2 |
| 32747155 | 1 | 2.17 | 1.99 | 2.36 | OR | Immunocompromised |  | Hernandez-Galdamez | Diego Rolando | Arch Med Res |  | hospitalization | 2 |
| 32747155 | 1 | 2.54 | 2.36 | 2.73 | OR | CKD |  | Hernandez-Galdamez | Diego Rolando | Arch Med Res |  | hospitalization | 2 |
| 32762106 | 1 | 2.60 | 1.77 | 3.83 | OR | CKD |  | Gottlieb | Michael | Acad Emerg Med |  | hospitalization | 2 |
| 32762106 | 1 | 1.77 | 1.46 | 2.16 | OR | HTN |  | Gottlieb | Michael | Acad Emerg Med |  | hospitalization | 2 |
| 32762106 | 1 | 1.69 | 1.44 | 1.98 | OR | Sex | male | Gottlieb | Michael | Acad Emerg Med |  | hospitalization | 2 |
| 32762106 | 1 | 4.55 | 3.40 | 6.09 | OR | Age | 65-74 | Gottlieb | Michael | Acad Emerg Med |  | hospitalization | 2 |
| 32762106 | 1 | 1.67 | 1.36 | 2.06 | OR | Age | 45-54 | Gottlieb | Michael | Acad Emerg Med |  | hospitalization | 2 |
| 32762106 | 1 | 0.92 | 0.60 | 1.43 | OR | Age | 0-18 | Gottlieb | Michael | Acad Emerg Med |  | hospitalization | 2 |
| 32762106 | 1 | 1.79 | 1.23 | 2.61 | OR | CHF |  | Gottlieb | Michael | Acad Emerg Med |  | hospitalization | 2 |
| 32762106 | 1 | 1.30 | 0.55 | 3.09 | OR | bloodborne cancer |  | Gottlieb | Michael | Acad Emerg Med |  | hospitalization | 2 |
| 32762106 | 1 | 0.68 | 0.25 | 1.84 | OR | HIV |  | Gottlieb | Michael | Acad Emerg Med |  | hospitalization | 2 |
| 32762106 | 1 | 0.82 | 0.65 | 1.04 | OR | Asthma |  | Gottlieb | Michael | Acad Emerg Med |  | hospitalization | 2 |
| 32762106 | 1 | 2.03 | 1.42 | 2.91 | OR | cirrhosis |  | Gottlieb | Michael | Acad Emerg Med |  | hospitalization | 2 |
| 32762106 | 1 | 0.75 | 0.54 | 1.05 | OR | Smoking | current | Gottlieb | Michael | Acad Emerg Med |  | hospitalization | 2 |
| 32762106 | 1 | 0.78 | 0.63 | 0.96 | OR | HLD |  | Gottlieb | Michael | Acad Emerg Med |  | hospitalization | 2 |
| 32762106 | 1 | 0.84 | 0.68 | 1.05 | OR | Smoking | former | Gottlieb | Michael | Acad Emerg Med |  | hospitalization | 2 |
| 32762106 | 1 | 1.62 | 0.93 | 2.82 | OR | COPD |  | Gottlieb | Michael | Acad Emerg Med |  | hospitalization | 2 |
| 32762106 | 1 | 1.99 | 1.58 | 2.51 | OR | Age | 55-64 | Gottlieb | Michael | Acad Emerg Med |  | hospitalization | 2 |
| 32762106 | 1 | 1.15 | 0.96 | 1.37 | OR | BMI | >=30 | Gottlieb | Michael | Acad Emerg Med |  | hospitalization | 2 |
| 32762106 | 1 | 2.22 | 1.12 | 4.41 | OR | ESRD |  | Gottlieb | Michael | Acad Emerg Med |  | hospitalization | 2 |
| 32762106 | 1 | 7.32 | 5.02 | 10.68 | OR | Age | 75+ | Gottlieb | Michael | Acad Emerg Med |  | hospitalization | 2 |
| 32762106 | 1 | 3.20 | 1.99 | 5.14 | OR | prior CVA |  | Gottlieb | Michael | Acad Emerg Med |  | hospitalization | 2 |
| 32762106 | 1 | 1.45 | 1.03 | 2.06 | OR | CAD |  | Gottlieb | Michael | Acad Emerg Med |  | hospitalization | 2 |
| 32762106 | 1 | 1.84 | 1.53 | 2.22 | OR | Diabetes |  | Gottlieb | Michael | Acad Emerg Med |  | hospitalization | 2 |
| 32762106 | 1 | 0.65 | 0.50 | 0.84 | OR | solid organ cancer |  | Gottlieb | Michael | Acad Emerg Med |  | hospitalization | 2 |
| 32762106 | 1 | 0.90 | 0.43 | 1.85 | OR | solid organ transplant |  | Gottlieb | Michael | Acad Emerg Med |  | hospitalization | 2 |
| 32762106 | 1 | 1.05 | 0.76 | 1.45 | OR | OSA |  | Gottlieb | Michael | Acad Emerg Med |  | hospitalization | 2 |
| 32803236 | 1 | 1.83 | 1.18 | 2.83 | OR | HTN |  | Zuniga-Moya | Julio | Clin Infect Dis |  | hospitalization | 2 |
| 32803236 | 1 | 2.55 | 1.66 | 3.92 | OR | Diabetes |  | Zuniga-Moya | Julio | Clin Infect Dis |  | hospitalization | 2 |
| 32803236 | 1 | 4.23 | 1.91 | 9.34 | OR | CHD |  | Zuniga-Moya | Julio | Clin Infect Dis |  | hospitalization | 2 |
| 32803236 | 1 | 4.05 | 2.85 | 5.76 | OR | Age | 45-69 | Zuniga-Moya | Julio | Clin Infect Dis |  | hospitalization | 2 |
| 32803236 | 1 | 9.12 | 5.24 | 15.86 | OR | Age | 70+ | Zuniga-Moya | Julio | Clin Infect Dis |  | hospitalization | 2 |
| 32803236 | 1 | 1.72 | 1.21 | 2.44 | OR | Sex | male | Zuniga-Moya | Julio | Clin Infect Dis |  | hospitalization | 2 |
| 32803236 | 1 | 5.17 | 1.54 | 17.27 | OR | COPD |  | Zuniga-Moya | Julio | Clin Infect Dis |  | hospitalization | 2 |
| 32803236 | 1 | 0.95 | 0.27 | 3.30 | OR | Age | 0-18 | Zuniga-Moya | Julio | Clin Infect Dis |  | hospitalization | 2 |
| 32803236 | 1 | 2.78 | 1.24 | 6.26 | OR | Asthma |  | Zuniga-Moya | Julio | Clin Infect Dis |  | hospitalization | 2 |
| 32803236 | 1 | 4.51 | 2.47 | 8.22 | OR | Obesity |  | Zuniga-Moya | Julio | Clin Infect Dis |  | hospitalization | 2 |
| 32853230 | 0 | 1.30 | 1.00 | 1.80 | HR | Age | 51-60 | Rossi | Paolo Giorgi | PLoS One |  | hospitalization + Mortality | 1 |
| 32853230 | 1 | 1.40 | 1.20 | 1.60 | HR | HTN |  | Rossi | Paolo Giorgi | PLoS One |  | hospitalization + Mortality | 1 |
| 32853230 | 1 | 1.40 | 1.10 | 1.70 | HR | Cancer |  | Rossi | Paolo Giorgi | PLoS One |  | hospitalization + Mortality | 1 |
| 32853230 | 1 | 1.90 | 1.30 | 2.90 | HR | CKD |  | Rossi | Paolo Giorgi | PLoS One |  | hospitalization + Mortality | 1 |
| 32853230 | 0 | 5.90 | 4.50 | 7.60 | HR | Age | 71-80 | Rossi | Paolo Giorgi | PLoS One |  | hospitalization + Mortality | 1 |
| 32853230 | 1 | 1.30 | 1.00 | 1.70 | HR | CHD |  | Rossi | Paolo Giorgi | PLoS One |  | hospitalization + Mortality | 1 |
| 32853230 | 1 | 1.50 | 1.30 | 1.90 | HR | Diabetes |  | Rossi | Paolo Giorgi | PLoS One |  | hospitalization + Mortality | 1 |
| 32853230 | 0 | 7.10 | 5.40 | 9.30 | HR | Age | >=81 | Rossi | Paolo Giorgi | PLoS One |  | hospitalization + Mortality | 1 |
| 32853230 | 0 | 3.20 | 2.40 | 4.10 | HR | Age | 61-70 | Rossi | Paolo Giorgi | PLoS One |  | hospitalization + Mortality | 1 |
| 32853230 | 1 | 1.90 | 1.40 | 2.50 | HR | COPD |  | Rossi | Paolo Giorgi | PLoS One |  | hospitalization + Mortality | 1 |
| 32853230 | 1 | 1.60 | 1.20 | 2.10 | HR | HF |  | Rossi | Paolo Giorgi | PLoS One |  | hospitalization + Mortality | 1 |
| 32853230 | 1 | 1.50 | 1.20 | 1.90 | HR | arrythmia |  | Rossi | Paolo Giorgi | PLoS One |  | hospitalization + Mortality | 1 |
| 32853230 | 1 | 1.40 | 0.90 | 2.00 | HR | Obesity |  | Rossi | Paolo Giorgi | PLoS One |  | hospitalization + Mortality | 1 |
| 32853230 | 1 | 1.20 | 0.90 | 1.80 | HR | Dementia |  | Rossi | Paolo Giorgi | PLoS One |  | hospitalization + Mortality | 1 |
| 32853230 | 1 | 1.20 | 0.80 | 1.80 | HR | vasc dis |  | Rossi | Paolo Giorgi | PLoS One |  | hospitalization + Mortality | 1 |
| 32853230 | 1 | 1.30 | 0.99 | 1.69 | HR | HLD |  | Rossi | Paolo Giorgi | PLoS One |  | hospitalization + Mortality | 1 |
| 32853230 | 0 | 1.40 | 1.20 | 1.60 | HR | Sex | male | Rossi | Paolo Giorgi | PLoS One |  | hospitalization + Mortality | 1 |
| 32887982 | 1 | 1.40 | 1.20 | 1.60 | OR | Cancer |  | Reilev | Mette | Int J Epidemiol |  | hospitalization | 2 |
| 32887982 | 1 | 1.80 | 1.60 | 2.00 | OR | Sex | Male | Reilev | Mette | Int J Epidemiol |  | hospitalization | 2 |
| 32887982 | 1 | 0.20 | 0.20 | 0.30 | OR | Age | 20-29 | Reilev | Mette | Int J Epidemiol |  | hospitalization | 2 |
| 32887982 | 1 | 2.10 | 1.80 | 2.50 | OR | Comorbidity | 2 | Reilev | Mette | Int J Epidemiol |  | hospitalization | 2 |
| 32887982 | 1 | 2.60 | 2.00 | 3.40 | OR | HF |  | Reilev | Mette | Int J Epidemiol |  | hospitalization | 2 |
| 32887982 | 1 | 3.10 | 2.50 | 3.80 | OR | Comorbidity | 3 | Reilev | Mette | Int J Epidemiol |  | hospitalization | 2 |
| 32887982 | 1 | 1.30 | 1.10 | 1.60 | OR | Stroke |  | Reilev | Mette | Int J Epidemiol |  | hospitalization | 2 |
| 32887982 | 1 | 1.60 | 1.30 | 1.90 | OR | Age | 60-69 | Reilev | Mette | Int J Epidemiol |  | hospitalization | 2 |
| 32887982 | 1 | 0.30 | 0.20 | 0.60 | OR | Age | 0-9 | Reilev | Mette | Int J Epidemiol |  | hospitalization | 2 |
| 32887982 | 1 | 0.20 | 0.10 | 0.30 | OR | Age | 10 to 19 | Reilev | Mette | Int J Epidemiol |  | hospitalization | 2 |
| 32887982 | 1 | 2.10 | 1.80 | 2.50 | OR | Obesity |  | Reilev | Mette | Int J Epidemiol |  | hospitalization | 2 |
| 32887982 | 1 | 4.70 | 3.90 | 5.70 | OR | Age | 70-79 | Reilev | Mette | Int J Epidemiol |  | hospitalization | 2 |
| 32887982 | 1 | 0.60 | 0.50 | 0.80 | OR | Age | 40-49 | Reilev | Mette | Int J Epidemiol |  | hospitalization | 2 |
| 32887982 | 1 | 0.40 | 0.30 | 0.50 | OR | Age | 30-39 | Reilev | Mette | Int J Epidemiol |  | hospitalization | 2 |
| 32887982 | 1 | 4.80 | 3.90 | 5.80 | OR | Age | 80-89 | Reilev | Mette | Int J Epidemiol |  | hospitalization | 2 |
| 32887982 | 1 | 2.90 | 2.20 | 3.90 | OR | CKD |  | Reilev | Mette | Int J Epidemiol |  | hospitalization | 2 |
| 32887982 | 1 | 1.70 | 1.50 | 1.90 | OR | HTN |  | Reilev | Mette | Int J Epidemiol |  | hospitalization | 2 |
| 32887982 | 1 | 1.80 | 1.50 | 2.10 | OR | Lung disease |  | Reilev | Mette | Int J Epidemiol |  | hospitalization | 2 |
| 32887982 | 1 | 1.00 |  |  | OR | Age | 50-59 | Reilev | Mette | Int J Epidemiol |  | hospitalization | 2 |
| 32887982 | 1 | 2.30 | 1.60 | 3.30 | OR | CLD |  | Reilev | Mette | Int J Epidemiol |  | hospitalization | 2 |
| 32887982 | 1 | 1.80 | 1.60 | 2.20 | OR | Diabetes |  | Reilev | Mette | Int J Epidemiol |  | hospitalization | 2 |
| 32887982 | 1 | 3.50 | 2.60 | 4.70 | OR | Age | >90 | Reilev | Mette | Int J Epidemiol |  | hospitalization | 2 |
| 32887982 | 1 | 1.40 | 1.20 | 1.70 | OR | Heart disease |  | Reilev | Mette | Int J Epidemiol |  | hospitalization | 2 |
| 32887982 | 1 | 1.40 | 1.20 | 1.70 | OR | AF |  | Reilev | Mette | Int J Epidemiol |  | hospitalization | 2 |
| 32887982 | 1 | 0.50 | 0.40 | 0.70 | OR | Dementia |  | Reilev | Mette | Int J Epidemiol |  | hospitalization | 2 |
| 32887982 | 1 | 3.90 | 3.20 | 4.80 | OR | Comorbidity | >4 | Reilev | Mette | Int J Epidemiol |  | hospitalization | 2 |
| 32887982 | 1 | 1.70 | 1.50 | 2.00 | OR | Comorbidity | 1 | Reilev | Mette | Int J Epidemiol |  | hospitalization | 2 |
| 32915872 | 1 | 2.05 | 1.81 | 2.32 | OR | Age | 50-74 | Carrillo-Vega | Maria | PLoS One |  | hospitalization | 2 |
| 32915872 | 1 | 1.54 | 1.37 | 1.74 | OR | Sex | Male | Carrillo-Vega | Maria | PLoS One |  | hospitalization | 2 |
| 32915872 | 1 | 3.84 | 2.90 | 5.10 | OR | Age | >75 | Carrillo-Vega | Maria | PLoS One |  | hospitalization | 2 |
| 32975575 | 0 | 1.38 | 0.79 | 2.43 | OR | Smoking | current or former | Munoz-Price | L Silvia | JAMA Netw Open |  | hospitalization | 2 |
| 32975575 | 0 | 0.99 | 0.71 | 2.10 | OR | BMI | unit increase | Munoz-Price | L Silvia | JAMA Netw Open |  | hospitalization | 2 |
| 32975575 | 0 | 1.78 | 1.00 | 3.18 | OR | Age | >=60 | Munoz-Price | L Silvia | JAMA Netw Open |  | hospitalization | 2 |
| 32975575 | 0 | 1.22 | 0.70 | 2.10 | OR | Sex | Male | Munoz-Price | L Silvia | JAMA Netw Open |  | hospitalization | 2 |
| 33084902 | 1 | 1.80 | 0.24 | 13.60 | OR | BMI | <18.5 | Gu | Tian | JAMA Netw Open |  | hospitalization | 2 |
| 33084902 | 1 | 1.00 | 0.59 | 1.68 | OR | CLD |  | Gu | Tian | JAMA Netw Open |  | hospitalization | 2 |
| 33084902 | 1 | 0.81 | 0.53 | 1.23 | OR | resp dis |  | Gu | Tian | JAMA Netw Open |  | hospitalization | 2 |
| 33084902 | 1 | 5.01 | 0.57 | 44.30 | OR | Age | <18 | Gu | Tian | JAMA Netw Open |  | hospitalization | 2 |
| 33084902 | 1 | 1.72 | 1.53 | 1.93 | OR | Age | per 10 yr incr | Gu | Tian | JAMA Netw Open |  | hospitalization | 2 |
| 33084902 | 1 | 0.75 | 0.32 | 1.77 | OR | Age | 35 to <50 | Gu | Tian | JAMA Netw Open |  | hospitalization | 2 |
| 33084902 | 1 | 1.82 | 1.25 | 2.64 | OR | Diabetes |  | Gu | Tian | JAMA Netw Open |  | hospitalization | 2 |
| 33084902 | 1 | 1.24 | 0.83 | 1.85 | OR | Immunocompromised |  | Gu | Tian | JAMA Netw Open |  | hospitalization | 2 |
| 33084902 | 1 | 2.43 | 1.38 | 4.30 | OR | BMI | >=30 | Gu | Tian | JAMA Netw Open |  | hospitalization | 2 |
| 33084902 | 1 | 0.72 | 0.20 | 2.53 | OR | Age | 50 to <65 | Gu | Tian | JAMA Netw Open |  | hospitalization | 2 |
| 33084902 | 1 | 2.87 | 1.87 | 4.42 | OR | CKD |  | Gu | Tian | JAMA Netw Open |  | hospitalization | 2 |
| 33084902 | 1 | 0.58 | 0.10 | 3.44 | OR | Age | 65 to <80 | Gu | Tian | JAMA Netw Open |  | hospitalization | 2 |
| 33084902 | 1 | 0.90 | 0.61 | 1.32 | OR | Cancer |  | Gu | Tian | JAMA Netw Open |  | hospitalization | 2 |
| 33084902 | 1 | 1.11 | 0.77 | 1.60 | OR | Smoking | ever | Gu | Tian | JAMA Netw Open |  | hospitalization | 2 |
| 33084902 | 1 | 1.21 | 0.83 | 1.77 | OR | Smoking | past | Gu | Tian | JAMA Netw Open |  | hospitalization | 2 |
| 33084902 | 1 | 0.51 | 0.17 | 1.52 | OR | Smoking | current | Gu | Tian | JAMA Netw Open |  | hospitalization | 2 |
| 33084902 | 1 | 1.04 | 1.01 | 1.06 | OR | BMI | per 1 unit incr | Gu | Tian | JAMA Netw Open |  | hospitalization | 2 |
| 33084902 | 1 | 1.91 | 1.36 | 2.68 | OR | Sex | male | Gu | Tian | JAMA Netw Open |  | hospitalization | 2 |
| 33084902 | 1 | 2.20 | 1.23 | 3.94 | OR | BMI | 25 to <30 | Gu | Tian | JAMA Netw Open |  | hospitalization | 2 |
| 33084902 | 1 | 1.35 | 0.87 | 2.08 | OR | circulatory disease |  | Gu | Tian | JAMA Netw Open |  | hospitalization | 2 |
| 33084902 | 1 | 1.15 | 0.10 | 13.10 | OR | Age | >=80 | Gu | Tian | JAMA Netw Open |  | hospitalization | 2 |
| 33180868 | 1 | 3.43 |  |  | OR | CKD |  | Oetjens | Matthew | PLoS One |  | hospitalization | 2 |
| 33180868 | 1 | 3.80 |  |  | OR | CHF |  | Oetjens | Matthew | PLoS One |  | hospitalization | 2 |
| 33180868 | 1 | 1.83 |  |  | OR | Diabetes |  | Oetjens | Matthew | PLoS One |  | hospitalization | 2 |
| 33180868 | 1 | 3.25 |  |  | OR | PVD |  | Oetjens | Matthew | PLoS One |  | hospitalization | 2 |
| 33334400 | 1 | 2.78 | 2.50 | 3.08 | OR | Age | 60-69 | Redondo-Bravo | Lidia | Euro Surveill |  | hospitalization + Mortality | 2 |
| 33334400 | 1 | 3.48 | 3.11 | 3.90 | OR | Age | >=80 | Redondo-Bravo | Lidia | Euro Surveill |  | hospitalization + Mortality | 2 |
| 33334400 | 1 | 1.40 | 1.28 | 1.53 | OR | HTN |  | Redondo-Bravo | Lidia | Euro Surveill |  | hospitalization + Mortality | 2 |
| 33334400 | 1 | 1.42 | 1.34 | 1.50 | OR | Sex | Male | Redondo-Bravo | Lidia | Euro Surveill |  | hospitalization + Mortality | 2 |
| 33334400 | 1 | 4.56 | 4.06 | 5.12 | OR | Age | 70-79 | Redondo-Bravo | Lidia | Euro Surveill |  | hospitalization + Mortality | 2 |
| 33334400 | 1 | 1.53 | 1.43 | 1.65 | OR | CVD |  | Redondo-Bravo | Lidia | Euro Surveill |  | hospitalization + Mortality | 2 |
| 33334400 | 1 | 1.46 | 1.33 | 1.59 | OR | Age | 40-59 | Redondo-Bravo | Lidia | Euro Surveill |  | hospitalization + Mortality | 2 |
| 33334400 | 1 | 1.66 | 1.52 | 1.82 | OR | Lung disease |  | Redondo-Bravo | Lidia | Euro Surveill |  | hospitalization + Mortality | 2 |
| 33334400 | 1 | 1.38 | 1.19 | 1.60 | OR | CKD |  | Redondo-Bravo | Lidia | Euro Surveill |  | hospitalization + Mortality | 2 |
| 33334400 | 1 | 1.38 | 1.27 | 1.50 | OR | Diabetes |  | Redondo-Bravo | Lidia | Euro Surveill |  | hospitalization + Mortality | 2 |
| 33354690 | 1 | 2.00 | 1.50 | 2.70 | OR | Diabetes |  | Chishinga, | Nathaniel | medRxiv |  | hospitalization + mortality | 2 |
| 33354690 | 1 | 5.00 | 2.90 | 8.60 | OR | Age | 55-64 | Chishinga, | Nathaniel | medRxiv |  | hospitalization + mortality | 2 |
| 33354690 | 1 | 2.70 | 1.80 | 4.20 | OR | Neurologic disease |  | Chishinga, | Nathaniel | medRxiv |  | hospitalization + mortality | 2 |
| 33354690 | 1 | 1.40 | 1.20 | 1.70 | OR | Sex | male | Chishinga, | Nathaniel | medRxiv |  | hospitalization + mortality | 2 |
| 33354690 | 1 | 12.80 | 7.10 | 22.90 | OR | Age | >=75 | Chishinga, | Nathaniel | medRxiv |  | hospitalization + mortality | 2 |
| 33354690 | 1 | 2.00 | 1.10 | 3.60 | OR | Age | 25-34 | Chishinga, | Nathaniel | medRxiv |  | hospitalization + mortality | 2 |
| 33354690 | 1 | 1.10 | 0.80 | 1.40 | OR | CVD |  | Chishinga, | Nathaniel | medRxiv |  | hospitalization + mortality | 2 |
| 33354690 | 1 | 0.60 | 0.20 | 1.90 | OR | CLD |  | Chishinga, | Nathaniel | medRxiv |  | hospitalization + mortality | 2 |
| 33354690 | 1 | 1.70 | 1.20 | 2.40 | OR | Lung disease |  | Chishinga, | Nathaniel | medRxiv |  | hospitalization + mortality | 2 |
| 33354690 | 1 | 2.10 | 1.20 | 3.60 | OR | Age | 35-44 | Chishinga, | Nathaniel | medRxiv |  | hospitalization + mortality | 2 |
| 33354690 | 1 | 3.90 | 2.40 | 6.30 | OR | CKD |  | Chishinga, | Nathaniel | medRxiv |  | hospitalization + mortality | 2 |
| 33354690 | 1 | 1.40 | 0.90 | 2.20 | OR | immunocompromised |  | Chishinga, | Nathaniel | medRxiv |  | hospitalization + mortality | 2 |
| 33354690 | 1 | 3.00 | 1.70 | 5.20 | OR | Age | 45-54 | Chishinga, | Nathaniel | medRxiv |  | hospitalization + mortality | 2 |
| 33354690 | 1 | 7.80 | 4.50 | 13.80 | OR | Age | 65-74 | Chishinga, | Nathaniel | medRxiv |  | hospitalization + mortality | 2 |
| 33461404 | 1 | 9.50 | 7.10 | 12.70 | RR | Age | 90+ | Telle | Kjetil | Scand J Public Health |  | mortality+hospitalization | 3 |
| 33461404 | 1 | 9.10 | 7.60 | 10.80 | RR | Age | 80-89 | Telle | Kjetil | Scand J Public Health |  | mortality+hospitalization | 3 |
| 33461404 | 1 | 4.00 | 3.40 | 4.70 | RR | Age | 60-69 | Telle | Kjetil | Scand J Public Health |  | mortality+hospitalization | 3 |
| 33461404 | 1 | 1.60 | 1.40 | 1.80 | RR | Comorbidity | yes | Telle | Kjetil | Scand J Public Health |  | mortality+hospitalization | 3 |
| 33461404 | 1 | 1.30 | 1.20 | 1.50 | RR | Sex | male | Telle | Kjetil | Scand J Public Health |  | mortality+hospitalization | 3 |
| 33461404 | 1 | 6.80 | 5.80 | 7.90 | RR | Age | 70-79 | Telle | Kjetil | Scand J Public Health |  | mortality+hospitalization | 3 |
| 33461404 | 1 | 2.50 | 2.10 | 2.90 | RR | Age | 50-59 | Telle | Kjetil | Scand J Public Health |  | mortality+hospitalization | 3 |

**pmid, PubMed ID**

**adj, adjusted (0=no, 1=yes)**

**lb, lower bound**

**ub, upper bound**

**est_meth, estimation method**

**cov_num, number of covariates adjusted for**
