# Supplementary material for: Risk factors for SARS-CoV-2 related mortality and hospitalization before vaccination: A meta-analysis
Source: PLOS Glob Public Health. 2022 Nov 2;2(11):e0001187. doi: 10.1371/journal.pgph.0001187 (PMC10021978; doi:10.1371/journal.pgph.0001187)
Supplement: S5 Table — (DOCX) [file pgph.0001187.s014.docx]

| **PMID** | **Study** | **Journal** | **Publication Year** | **Publication Month** | **Population Size** | **Country** | **City/State** | **Population Type** | **Study Design** | **Study Sites** | **Outcome** | **Case Identification** | **Months Studied** |
| --- | --- | --- | --- | --- | --- | --- | --- | --- | --- | --- | --- | --- | --- |
| 32444366 | Petrilli, et al. | BMJ Clinical Research ed. | 2020 | May | 5279 | United States | New York City, New York | general | prospective | multi | mortality + hospitalization | lab | March-April 2020 |
| 32470210 | Hajifathalian , et al. | Obesity | 2020 | May | 770 | United States | New York City, New York | hospitalized | retrospective | multi | mortality | lab | March-April 2020 |
| 32474598 | Bello-Chavolla, et al | The Journal of clinical endocrinology and metabolism | 2020 | May | 177133 | Mexico |  | general | not listed | multi | mortality | lab | to May 2020 |
| 32487789 | Escalera-Antezana, et al. | Le infezioni in medicina | 2020 | June | 107 | Bolivia |  | general | retrospective | multi | mortality | lab | March, 2020 |
| 32496252 | Tambe, et al. | Indian J Pub Health | 2020 | June | 197 | India | Pune, Maharashtra | hospitalized | cross-sectional | single | mortality | lab | March-April 2020 |
| 32496259 | Mishra, et al. | Indian J Public Health | 2020 | June | 445 | India | Karnataka | hospitalized | retrospective | multi | mortality | lab | March-April 2020 |
| 32537662 | Rath, et al. | Clin Res Cardiol | 2020 | June | 123 | Germany | Tübingen, Baden-Württemberg | hospitalized | prospective | single | mortality | lab | February-March 2020 |
| 32546725 | Chen, et al. | Leukemia | 2020 | June | 1859 | China | Wuhan | hospitalized | not listed | multi | mortality | lab | January-April 2020 |
| 32589784 | Pettit, et al. | Obesity | 2020 | August | 238 | United States | Chicago, Illinois | hospitalized | retrospective | single | mortality | lab | March-April 2020 |
| 32607513 | Mendy, et al. | medRxiv | 2020 | June | 689 | United States | Cincinnatti, Ohio | general | retrospective | single | mortality + hospitalization | lab | March-May 2020 |
| 32620056 | Shah, et al. | Ann Med | 2020 | July | 522 | United States | Albany, Georgia | hospitalized | retrospective | multi | mortality | lab | March-May 2020 |
| 32640463 | Williamson, et al. | Nature | 2020 | July | 17278392 | England |  | general | quantitative cohort | multi | mortality | clinical | February-May 2020 |
| 32710646 | Harmouch, et al. | Journal Med Virol | 2020 | August | 560 | United States | Bethlehem, Pennsylvania | hospitalized | retrospective | single | mortality | lab | March-April 2020 |
| 32712623 | Nakeshbandi, et al. | Int J Obes | 2020 | July | 504 | United States | Brooklyn, New York | hospitalized | retrospective | single | mortality | lab | March-April 2020 |
| 32722159 | Nogueira, et al. | J Clin Med | 2020 | July | 20293 | Portugal |  | general | not listed | multi | mortality + hospitalization | lab | January-April 2020 |
| 32726242 | Smith, et al. | West J Emerg Med | 2020 | July | 346 | United States | Waterbury, Connecticut Hartford, Connecticut Stafford Connecticut Springfield, Massachusetts | hospitalized | retrospective | multi | mortality | lab | March-April 2020 |
| 32730358 | Zhao, et al. | PLoS One | 2020 | July | 641 | United States | Stony Brook, New York | hospitalized | retrospective | single | mortality | lab | March-April 2020 |
| 32743602 | McPadden, et al. | medRxiv | 2020 | July | 28605 | United States | New Haven, Connecticut | general | retrospective | multi | mortality + hospitalization | lab | March-April 2020 |
| 32744714 | De Vito, et al | Eur Rev Med Pharmacol Sci | 2020 | July | 87 | Italy | Sardinia | hospitalized | retrospective | single | mortality | lab | March-April 2020 |
| 32766546 | Almazeedi, et al. | EClinicalMedicine | 2020 | July | 1096 | Kuwait |  | hospitalized | retrospective | single | mortality | lab | February-April 2020 |
| 32783686 | Tartof, et al. | Ann Intern Med | 2020 | August | 6916 | United States | Southern California | general | retrospective | multi | mortality | diagnostic code or lab | February-May 2020 |
| 32810610 | Vena, et al. | Clin Microbiol Infect | 2020 | August | 317 | Italy | Genoa, Liguria | hospitalized | retrospective | single | mortality | lab | February-March 2020 |
| 32815621 | Czernichow, et al. | Obesity | 2020 | November | 4056 | France | Paris | hospitalized | prospective | multi | mortality | lab | February-April 2020 |
| 32853230 | Rossi, et al. | PLoS One | 2020 | August | 2653 | Italy | Reggio Emilia | general | prospective | multi | mortality + hospitalization | lab | February-April 2020 |
| 32887982 | Reilev, et al. | Int J Epidemiol | 2020 | October | 11122 | Denmark |  | general | retrospective | multi | mortality + hospitalization | lab | February-May 2020 |
| 32892789 | Santos, et al. | Epidemiol Infect | 2020 | September | 46285 | Brazil |  | hospitalized | retrospective | multi | mortality | lab | February-June 2020 |
| 32945856 | Miller, et al. | Clin Infect Dis | 2020 | September | 3633 | United States | Detroit, Michigan | presenting to  emergency departments | retrospective | multi | mortality | lab | March-April 2020 |
| 32960645 | Garibaldi, et al. | Ann Intern Med | 2021 | January | 832 | United States | Baltimore, Maryland Columbia, Maryland  Bethesda, Maryland Washington D.C. | hospitalized | retrospective | multi | mortality | lab | March-June 2020 |
| 32975574 | Kabarriti, et al. | JAMA Netw Open | 2020 | September | 9268 | United States | Bronx, New York | general | cohort | single | mortality | lab | March-April 2020 |
| 32975575 | Munoz-Price, et al. | JAMA Netw Open | 2020 | September | 2595 | United States | Milwaukee, Wisconsin | general | cross-sectional | single | mortality + hospitalization | lab | March, 2020 |
| 33020114 | Adrish, et al. | BMJ Open Respir Res | 2020 | October | 1173 | United States | Bronx, New York | hospitalized | retrospective | multi | mortality | lab | March-May 2020 |
| 33020335 | Rozaliyani, et al. | Indones J Intern Med | 2020 | July | 4052 | Indonesia |  | general | retrospective | multi | mortality | lab | March-April 2020 |
| 33035307 | Munblit, et al. | Clin Infect Dis | 2020 | October | 3480 | Russia | Moscow | hospitalized | observational | multi | mortality | lab or clinical | April-May 2020 |
| 33038592 | Calmes, et al. | J Allergy Clin Immunol Pract | 2020 | October | 596 | Belgium | Leige | hospitalized | not listed | single | mortality | lab | March-April 2020 |
| 33043705 | Ramachandran, et al. | Cancer Control | 2020 | October | 725 | United States | Brooklyn, New York | hospitalized | retrospective | single | mortality | lab | March-April 2020 |
| 33051749 | Mohammed, et al. | J Racial Ethn Health Disparities | 2020 | October | 1028 | Africa |  | general | cross-sectional | multi | mortality | lab | August, 2020 |
| 33090436 | Doganci, et al. | Eur Rev Med Pharmacol Sci | 2020 | October | 397 | Turkey | Ankara | hospitalized | retrospective | single | mortality | lab | March-May 2020 |
| 33112411 | Lunski, et al. | Cancer | 2020 | October | 4760 | United States | Louisiana | general | retrospective | multi | mortality | lab | March-April 2020 |
| 33115547 | Islam, et al. | Epidemiol Infect | 2020 | October | 1016 | Bangladesh | Dhaka | general | retrospective | multi | mortality | lab | March-June 2020 |
| 33128848 | Kim, et al. | Obesity | 2020 | October | 10861 | United States | Long Island, New York City, Weschester New York | hospitalized | not listed | multi | mortality | lab | March-April 2020 |
| 33130213 | Tehrani, et al. | Int J Infect Dis | 2020 | October | 255 | Sweden | Stockholm | hospitalized | retrospective | single | mortality | lab | March-April 2020 |
| 33134966 | Zali, et al. | Arch Acad Emerg Med | 2020 | September | 16016 | Iran | Tehran | hospitalized | cross-sectional | multi | mortality | lab or radiologic | February-May 2020 |
| 33141353 | Farrell, et al. | Ir J Med sci | 2020 | November | 257 | Ireland |  | hospitalized | not listed | single | mortality | lab | March-May 2020 |
| 33169090 | Shah, et al. | Ann Med Surg | 2020 | November | 487 | United States | New Jersey | hospitalized | retrospective | single | mortality | lab | January-May 2020 |
| 33172229 | Ayaz, et al. | Acute Crit Care | 2020 | November | 66 | Pakistan |  | hospitalized | retrospective | single | mortality | lab | March-April 2020 |
| 33173851 | Rodriguez-Nava, et al. | Mayo Clin Proc Innov Qual Outcomes | 2020 | November | 313 | United States | Cook County, Illinois | hospitalized | retrospective | single | mortality | lab | March-May 2020 |
| 33218161 | Lee, et al. | Int J Environ Res Public Health | 2020 | November | 7339 | Korea |  | general | retrospective | multi | mortality | lab | until May 2020 |
| 33229434 | Loffi, et al. | Open Heart | 2020 | November | 1252 | Italy | Lombary region | hospitalized | retrospective | single | mortality | (clinical and radiologic) or lab | February-March 2020 |
| 33246431 | van Halem, et al. | BMC Infect Dis | 2020 | November | 319 | Belgium | Hasselt | hospitalized | retrospective | single | mortality | lab | March - Apr 2020 |
| 33272355 | Kaeuffer, et al. | Euro Surveill | 2020 | December | 1045 | France | Alsace (Strasbourg and Mulhouse) | hospitalized | prospective | multi | mortality | lab | March, 2020 |
| 33278893 | Alguwaihes, et al. | Cardiovasc Diabetol | 2020 | December | 439 | Saudi Arabia |  | hospitalized | retrospective | single | mortality | lab | May-July 2020 |
| 33298991 | Rechtman, et al. | Sci Rep | 2020 | December | 8770 | United States | New York City, New York | general | retrospective | multi | mortality | lab | to April 2020 |
| 33324068 | Omar, et al. | J Multidiscip Health | 2020 | December | 88 | Eastern Sudan |  | hospitalized | retrospective | single | mortality | lab | April-July 2020 |
| 33331576 | Caliskan, et al. | Rev Assoc Med Bras | 2020 | December | 813 | Turkey | Istanbul | hospitalized | retrospective | single | mortality | lab or clinical or radiographic | March-May 2020 |
| 33333477 | Moradi, et al. | Am J Emerg Med | 2020 | December | 219 | Iran | Mashhad, Razavi Khorasan | hospitalized | retrospective | single | mortality | lab | March-June 2020 |
| 33334400 | Redondo-Bravo, et al. | Euro Surveill | 2020 | December | 218652 | Spain |  | general | not listed | multi | mortality + hospitalization | lab | January-April 2020 |
| 33334842 | Elimian, et al. | BMJ Open | 2020 | December | 35567 | Nigeria |  | general | retrospective | multi | mortality | lab | Febuary-June 2020 |
| 33338063 | Matangila | PLoS One | 2020 | December | 160 | Democratic Republic of Congo | Kinshasa | hospitalized | retrospective | single | mortality | lab | March-July 2020 |
| 33354690 | Chishinga, et al. | medRxiv | 2020 | December | 1969:mortality, 2820:hospitalization | United States | Atlanta, Georgia | general | retrospective | multi | mortality + hospitalization | lab | March-May 2020 |
| 33370364 | Mejia, et al. | PLoS One | 2020 | December | 369 | Peru | Lima | hospitalized | retrospective | single | mortality | (clinical and radiologic) or lab | March-June 2020 |
| 33390322 | McNeill, et al. | Obes Res Clin Pract | 2020 | December | 781 | United States | Boston, Massachusetts | hospitalized | observational | single | mortality | lab | February-April 2020 |
| 33393318 | Javanian, et al. | Bratisl Med J | 2021 | January | 557 | Iran | Babol, Mazandaran | hospitalized | retrospective | multi | mortality | lab | March-April 2020 |
| 33395425 | Ortiz-Prado | PLoS Negl Trop Dis | 2021 | January | 9469 | Ecuador |  | general | cross-sectional | multi | mortality | lab | February-April 2020 |
| 33409033 | Rustgi, et al. | Cureus | 2020 | November | 403 | United States | New Brunswick, New Jersey | hospitalized | retrospective | single | mortality | lab | January-April 2020 |
| 33410296 | Kristic, et al. | Croat Med J | 2020 | December | 17206 | Croatia |  | general | not listed | multi | mortality | lab | March-September 2020 |
| 33444432 | Saurabh, et al. | Trans R Soc Trop Med Hyg | 2021 | January | 911 | India | Rajasthan | hospitalized | prospective | single | mortality | lab | March-July 2020 |
| 33449333 | Salari, et al. | Ir J Med Sci | 2021 | January | 103 | Iran | Rasht, Guilan | hospitalized | case control | single | mortality | lab and clinical and radiologic | April-August 2020 |
| 33468081 | Rastad, et al | BMC Nephrol | 2021 | January | 520 | Iran | Alborz | hospitalized | retrospective | not listed | mortality | lab or radiologic | February-April 2020 |
| 33500292 | Mash, et al. | BMJ Open | 2021 | January | 1376 | South Africa | Western Cape | general | cross-sectional | multi | mortality | lab | March-June 2020 |
| 33519709 | Crouse, et al. | Front Endocrinol | 2021 | January | 25326 | United States | Birmingham, Alabama | general | retrospective | single | mortality | lab | February-June 2020 |
| 33528146 | Kvale, et al. | Tidsskr Nor Laegeforen | 2020 | December | 8809 | Norway |  | general | registry analysis | multi | mortality | lab | "first half 2020" |
| 32437224 | Azar, et al. | Health Affairs | 2020 | May | 1052 | United States | Northern California | general | retrospective | multi | hospitalization | lab | January-April 2020 |
| 32459916 | Price-Haywood, et al. | NEJM | 2020 | June | 3626 | United States | Louisiana | general | retrospective | multi | hospitalization | lab | March-April 2020 |
| 32497776 | Lassale, et al. | Brain Behav Immun | 2020 | June | 340966 | England |  | general | prospective | multi | hospitalization | lab | March - April 2020 |
| 32522462 | Zhu, et al. | J Allergy Clin Immunol | 2020 | June | 492768 | England |  | general | prospective | multi | hospitalization | lab | March - April 2020 |
| 32682453 | Soares, et al. | Am J Trop Med Hyg | 2020 | July | 10713 | Brazil | Espirito Santo | general | retrospective | multi | hospitalization | lab or clinical | Febryary-June 2020 |
| 32700398 | Merzon, et al. | FEBS J | 2020 | August | 7807 | Israel |  | general | population based | multi | hospitalization | lab | February-April 2020 |
| 32747155 | Hernandez-Galdamez, et al. | Arch Med Res | 2020 | July | 211003 | Mexico |  | general | cross-sectional | multi | hospitalization | lab | to June 2020 |
| 32762106 | Gottlieb, et al. | Acad Emerg Med | 2020 | August | 8673 | United States | Chicago, Illinois | general | retrospective | single | hospitalization | lab | March-June 2020 |
| 32803236 | Zuniga-Moya, et al. | Clin Infect Dis | 2020 | August | 877 | Honduras | San Pedro Sula | general | not listed | multi | hospitalization | lab | March-May 2020 |
| 32915872 | Carrillo-Vega, et al. | PLoS One | 2020 | September | 10544 | Mexico |  | general | retrospective | multi | hospitalization | lab | to April 2020 |
| 33084902 | Gu, et al. | JAMA Netw Open | 2020 | October | 5698 | United States | Ann Arbor, Michigan | general | retrospective | multi | hospitalization | lab | March-April 2020 |
| 33180868 | Oetjens, et al. | PLoS One | 2020 | November | 12971 | United States | Pennsylvania | general | not listed | multi | hospitalization | lab | March-May 2020 |
| 33461404 | Telle, et al. | Scand J Public Health | 2021 | January | 8569 | Norway |  | general | prospective | multi | hospitalization | lab | January to June 2020 |
