# Supplementary material for: Risk factors for SARS-CoV-2 related mortality and hospitalization before vaccination: A meta-analysis
Source: PLOS Glob Public Health. 2022 Nov 2;2(11):e0001187. doi: 10.1371/journal.pgph.0001187 (PMC10021978; doi:10.1371/journal.pgph.0001187)
Supplement: Attachment — Submitted filename: Response to Reviewers.docx [file pgph.0001187.s016.docx]

We thank the reviewers for the thorough review of our manuscript. We have addressed the comments as follows and highlighted modifications in the manuscript with track changes.

*Journal Requirements:*

*1. Please provide separate figure files in .tif or .eps format and remove the embedded figure from the manuscript file.*

**Response:** We have removed the embedded figures from the manuscript and have included as separate figure (.tif) files.

*2. We notice that your supplementary [figures/tables] are included in the manuscript file. Please remove them and upload them with the file type 'Supporting Information'. Please ensure that each Supporting Information file has a legend listed in the manuscript after the references list.*

**Response:** We have removed the supplementary information from the manuscript file and have included them in the ‘Supporting Information’ file. All Supporting Information files now have a legend after the references list.

*3. Please review your reference list to ensure that it is complete and correct. If you have cited papers that have been retracted, please include the rationale for doing so in the manuscript text or remove these references and replace them with relevant current references. Any changes to the reference list should be mentioned in the rebuttal letter that accompanies your revised manuscript. If you need to cite a retracted article, indicate the article’s retracted status in the References list and also include a citation and full reference for the retraction notice.*

**Response:** The reference list has been checked for complete and correct information.


*Reviewers' comments:*

*5. Review Comments to the Author

Please use the space provided to explain your answers to the questions above. You may also include additional comments for the author, including concerns about dual publication, research ethics, or publication ethics. (Please upload your review as an attachment if it exceeds 20,000 characters)*

*Reviewer #1: The authors presented a systematic review with meta-analysis, from studies published between January 2020 to January 2021, before the introduction of the vaccine. The article presents the “Risk factors for SARS-CoV-2 related mortality and hospitalization”.
It is suggested that the title of the article include 'before vaccination': Risk factors for SARS-CoV-2 related mortality and hospitalization before vaccination: a meta-analysis
The selected data, based on the Systematic Review methodology, present adequate stages.
This article reveals important data for the public health scenario, in which men and people with some medical condition, such as kidney, heart and lung diseases were at greater risk for mortality or hospitalization due to COVID-19.*

**Response:** We thank the reviewer for the comment on the title. We have now modified the title to “Risk factors for SARS-CoV-2 related mortality and hospitalization before vaccination: a meta-analysis”.

*Reviewer #2: Review for PGPH-D-22-00926
Risk factors for SARS-CoV-2 related mortality and hospitalization: a meta-analysis

I appreciate the invitation to review this manuscript that addresses an important public health issue. The manuscript mainly investigates risk factors for mortality and hospitalization, estimates individual risk factor contribution, and determines drivers of published estimate variances.

The manuscript is well written and understandable. It is technically sound, and the data do support the conclusions. The statistical analysis been performed appropriately and rigorously.

I have only one note that refers to the last part of the aim of the study, which was not fully met. Therefore, I suggest that the authors review the study aim and discuss more robustly the drivers of published estimate variances.*

**Response:** We thank the reviewer for the comments. The last aim of the study was to determine drivers of point estimate effect size variances. We have shown studies from Europe and Asia tend to have a higher likelihood of reporting smaller effect sizes of male sex on hospitalization. We also demonstrated that studies conducted in North America and Europe are more likely to report smaller effect sizes of CKD as a risk factor for mortality. Additionally, we found retrospective studies to be more likely to report smaller effect sizes attributed to CHF. In terms of geographic variation, these differences *could* be related to varying lifestyles and treatment modalities from region to region, although not proven. We have now included a more clear and robust description in our discussion section.

*Reviewer #4: The manuscript is methodologically robust. The data support the conclusions presented but no substantially new information is presented. My impression is that figures 1, 2, 3, 4 and 5 are presented in duplicate and supplementary figures are not available.*

**Response:** We thank the reviewer for this comment. Figure 1 shows the flow diagram for study inclusion in the meta-analysis. Figures 2, 3, and 5 display the meta-analysis for each risk factor for mortality and hospitalization. Figure 4 shows the stratified meta-analysis for chronic kidney disease by study region. We have ensured that none of the figures have been presented in duplicate.

We also appreciate the comment about the supplementary information. We have now included a legend for each supplementary file after the references.
